# Saturated palmitic acid induces myocardial inflammatory injuries through direct binding to TLR4 accessory protein MD2

**DOI:** 10.1038/ncomms13997

**Published:** 2017-01-03

**Authors:** Yi Wang, Yuanyuan Qian, Qilu Fang, Peng Zhong, Weixin Li, Lintao Wang, Weitao Fu, Yali Zhang, Zheng Xu, Xiaokun Li, Guang Liang

**Affiliations:** 1 Chemical Biology Research Center, School of Pharmaceutical Sciences, Wenzhou Medical University, Wenzhou, Zhejiang 325035, China

## Abstract

Obesity increases the risk for a number of diseases including cardiovascular diseases and type 2 diabetes. Excess saturated fatty acids (SFAs) in obesity play a significant role in cardiovascular diseases by activating innate immunity responses. However, the mechanisms by which SFAs activate the innate immune system are not fully known. Here we report that palmitic acid (PA), the most abundant circulating SFA, induces myocardial inflammatory injury through the Toll-like receptor 4 (TLR4) accessory protein MD2 in mouse and cell culture experimental models. *Md2* knockout mice are protected against PA- and high-fat diet-induced myocardial injury. Studies of cell surface binding, cell-free protein–protein interactions and molecular docking simulations indicate that PA directly binds to MD2, supporting a mechanism by which PA activates TLR4 and downstream inflammatory responses. We conclude that PA is a crucial contributor to obesity-associated myocardial injury, which is likely regulated via its direct binding to MD2.

## Abstract (web summary)

The free fatty acid-mediated inflammatory activities are regulated through TLR4. Here the authors show that palmitic acid binds to MD2, initiating complex formation with TLR4, recruitment of MyD88, and subsequent activation of pro-inflammatory molecules, and that MD2 blockade protects against diet-induced cardiac dysfunction.

Obesity is a global epidemic^1^ and is associated with increased risk of developing cardiovascular diseases^2^. Various aspects of cardiac tissue remodelling are clearly linked to obesity and include cardiac fibrosis and cardiomyocyte hypertrophy^3^,^4^. Although the pathophysiology of obesity-related cardiac damage is complex and multifaceted, inflammation is believed to be crucial^5^,^6^. It is also known that free fatty acid (FFA) levels are increased in obese subjects^7^,^8^. Elevated levels of FFAs are independently associated with greater risks of cardiovascular events^9^,^10^,^11^. Among circulating FFAs, saturated fatty acid (SFA) palmitate (C16:0) is one of the most abundant^12^ and is increased in obese children and adolescents^13^. Studies have also established that SFAs activate inflammatory and innate immune responses^14^,^15^,^16^,^17^,^18^. We^19^ and others^20^,^21^ found that palmitic acid (PA) induces an inflammatory phenotype in cardiomyocytes. This inflammatory activity is characterized by increased production of pro-inflammatory cytokines and oxidants, leading to cellular hypertrophy and apoptosis. The findings indicate that elevated levels of PA and likely other SFAs can contribute significantly to cardiac damage.

Toll-like receptor 4 (TLR4) is an essential modulator of innate immunity, and links innate immunity and metabolic disorders including obesity^14^,^16^,^22^,^23^. The signalling mechanism engaged by SFAs appears to be through TLR4, triggering acute and chronic inflammation^14^,^15^,^16^,^22^,^24^,^25^. Studies have shown that a SFA- but not unsaturated fatty acid-rich diet induces leptin resistance, TLR4 activation and endoplasmic reticulum stress^26^. Furthermore, TLR4 blockade suppresses PA-induced cytokine production^22^. To date, it remains an open question as to how SFAs (as well as other metabolic factors) activate TLR4-dependent innate immune responses.

TLR4 is a pattern recognition receptor, and versatile in its ability to bind a spectrum of ligands related to infectious agents to elicit innate immune responses^27^. The transduction mechanism for TLR4 activation is well characterized for the Gram-negative bacterial product, lipopolysaccharide (LPS). For the LPS response, TLR4 activation requires complex formation with an accessory protein called myeloid differentiation protein 2 (MD2). MD2 is an extracellular molecule indispensable for LPS recognition. Binding of lipid A of LPS with MD2 leads to recruitment of adaptor protein MyD88 and production of a host of pro-inflammatory molecules^28^,^29^. Growing evidence indicates that TLR4 is crucial not only for LPS-mediated inflammatory responses but also for non-microbial ligands such as SFAs^14^,^27^. However, it is not clear whether SFAs engage similar transduction processes involving TLR4 and activating innate immunity in obesity-related cardiac injury. Based on the current understanding of binding of saturated lipid chains of LPS within the MD2 hydrophobic pocket^30^, we postulated that SFAs interact with MD2 by a similar mechanism.

We investigated the overall hypothesis that PA (and possibly other SFAs) drives the development of myocardial injury through a mechanism of direct interactions with MD2. Results indicate that the PA- and (HFD)-induced myocardial inflammatory injury is dependent on MD2, the dependency likely attributed to direct PA binding. The findings provide new mechanistic insight linking FFAs in obsesity and TLR4-mediated immunity in cardiovascular diseases.

## Results

### PA induces inflammation in the heart through MD2

**Figure 1:**
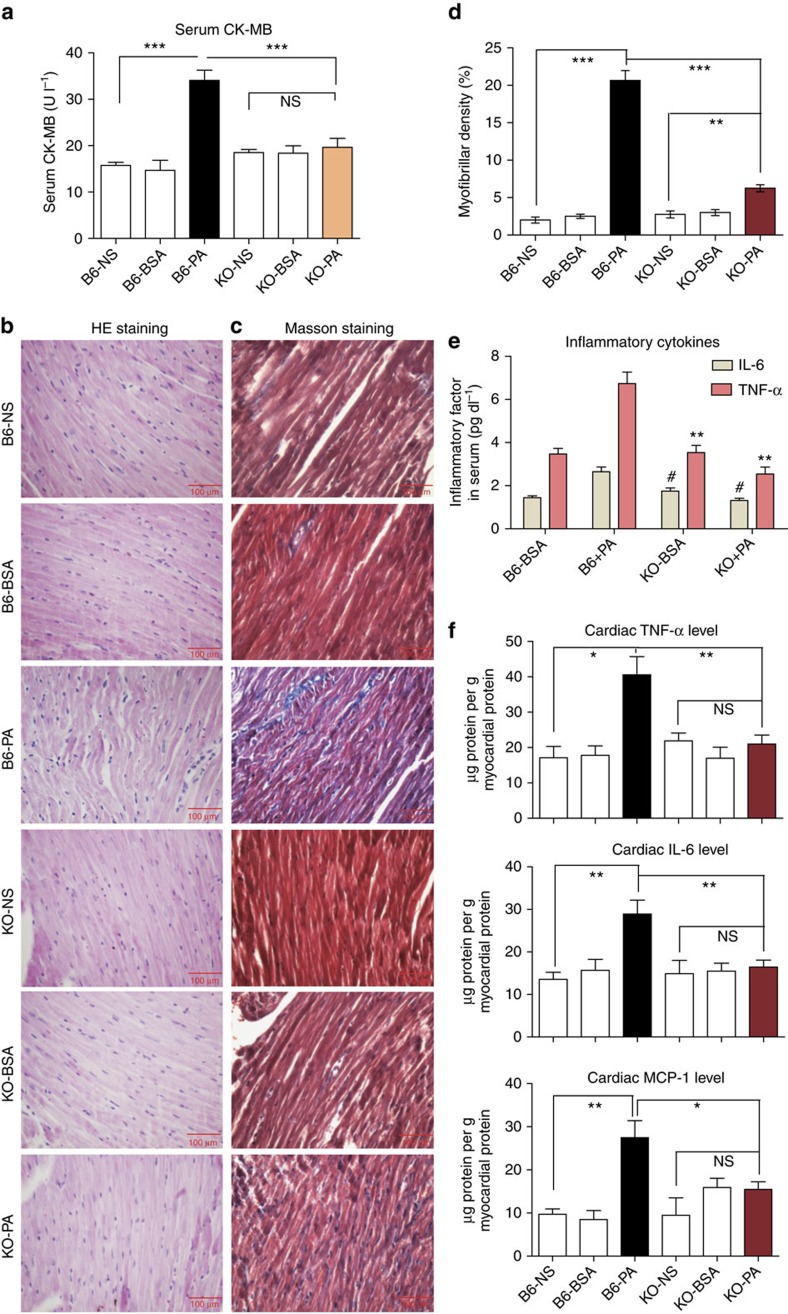
*Md2* knockout protects against PA-induced myocardial injury. Wild-type (B6) or *Md2*^*−/−*^ mice (KO) were challenged with intravenous (i.v.) injection of 5 mM PA, normal saline (NS) or 5% BSA (vehicle control) twice daily for 7 days; *n*=8. (**a**) Serum CK-MB values reported in U l^−1^. (**b**) Representative micrographs of heart tissue morphology showing hematoxylin- and eosin-stained sections; scale bars, 100 μm. (**c**) Representative micrographs of histochemical staining for connective tissue using Masson’s trichrome; scale bars=100 μM. (**d**) Quantification of trichrome staining of heart tissues of PA-challenged mice. (**e**) Serum TNF-α and IL-6 protein levels (**P<0.01, ^#^*P*<0.01, compared with B6+PA group). (**f**) Protein levels of TNF-α, IL-6 and MCP-1 in heart tissue reported as μg g^−1^ myocardial protein. Data in **a** and **d**–**f** are reported as mean±s.e.m. and analysed by Student’s *t*-test, *P<0.05, **P<0.01, ***P<0.001, B6-PA group compared with either controls or KO groups; NS, not significant.

We first determined whether PA induces innate immune responses in the cardiac tissue and whether MD2 is involved in this process. We injected PA in wild-type B6 and *Md2*^*−/−*^ knockout mice for 7 days and examined the heart tissues. Our results show that PA induced a significant increase in serum creatine kinase MB (CK-MB) in C57BL/6 (B6) mice (Fig. 1a). Elevated CK-MB is indicative of significant cardiac damage in the wild-type mice. Indeed, we observed myofiber disorganization (Fig. 1b) and pronounced collagen deposition in the heart tissues of PA-challenged mice compared with normal saline (NS)- or bovine serum albumin (BSA)-injected control mice (Fig. 1c,d; Supplementary Fig. 1a). However, such injuries were not seen in the *Md2* knockout (KO) mice (Fig. 1a–d; Supplementary Fig. 1a), indicating that *Md2* deficiency was protective against PA-induced cardiac damage.

We then examined whether PA-induced cardiac damage was associated with an inflammatory response. Our results show that PA increased serum levels of pro-inflammatory cytokines tumour necrosis factor-α (TNF-α) and interleukin (IL)-6 (Fig. 1e). The levels of these cytokines were also increased in the cardiac tissues (Fig. 1f; Supplementary Fig. 1b–e). In addition, we observed increased cleaved caspase-3 and terminal deoxynucleotidyl transferase-mediated FITC-dUTP nick end labelling (TUNEL)-positive apoptotic cells in the cardiac tissues (Supplementary Fig. 2c–e). We further analysed the expression of monocyte chemoattractant protein 1 (MCP-1) and intracellular adhesion molecule 1 (ICAM-1) in the cardiac tissues as additional indicators of local inflammatory response activation. IL-6 is known to increase both MCP-1 and ICAM-1 expression^31^ and facilitate monocyte recruitment. In the wild-type mice, PA increased expression of MCP-1 and ICAM-1 (Fig. 1f; Supplementary Fig. 1f), indicating activation of local inflammatory processes. However, PA did not increase expression of these inflammatory molecules in the KO mice. Supplementary Fig. 1g–h provides additional supportive data of the protective effects of *Md2* deletion against PA-induced cardiac fibrosis, as indicated by reduced messenger RNA (mRNA) expression of fibrogenic factors, transforming growth factor-β (TGF-β) and collagen 1.

These studies indicated that an acute infusion and elevation of PA was sufficient to induce myocardial injury. We confirmed the model of myocardial injury with acute PA infusion by repeating the mouse study twice using the same protocol. We observed increased levels of serum lactate dehydrogenase (LDH) and CK-MB (Supplementary Fig. 2a–b), which was consistent with pronounced damage. These results clearly support the notion that PA challenge caused significant cardiac damage in mice.

### PA induces MD2-dependent inflammatory cardiomyocyte injury

Since significant myocardial injury was associated with effects of PA, we tested whether MD2 blockade in cardiomyocytes was protective against PA challenge. For these studies, we used H9C2 cardiomyocyte-like cell line to delineate the effects of PA and confirm the results using primary neonatal rat cardiomyocytes. To functionally inhibited MD2 in this system, we used a small-molecule inhibitor of MD2 (L6H9; Supplementary Fig. 3a) and *Md2* short interfering RNA (siRNA) transfection. We have previously characterized the anti-inflammatory activity of L6H9 in LPS-stimulated macrophages^32^ and report that L6H9 selectively inhibits the activity of MD2 (Supplementary Fig. 3b–h).

**Figure 2:**
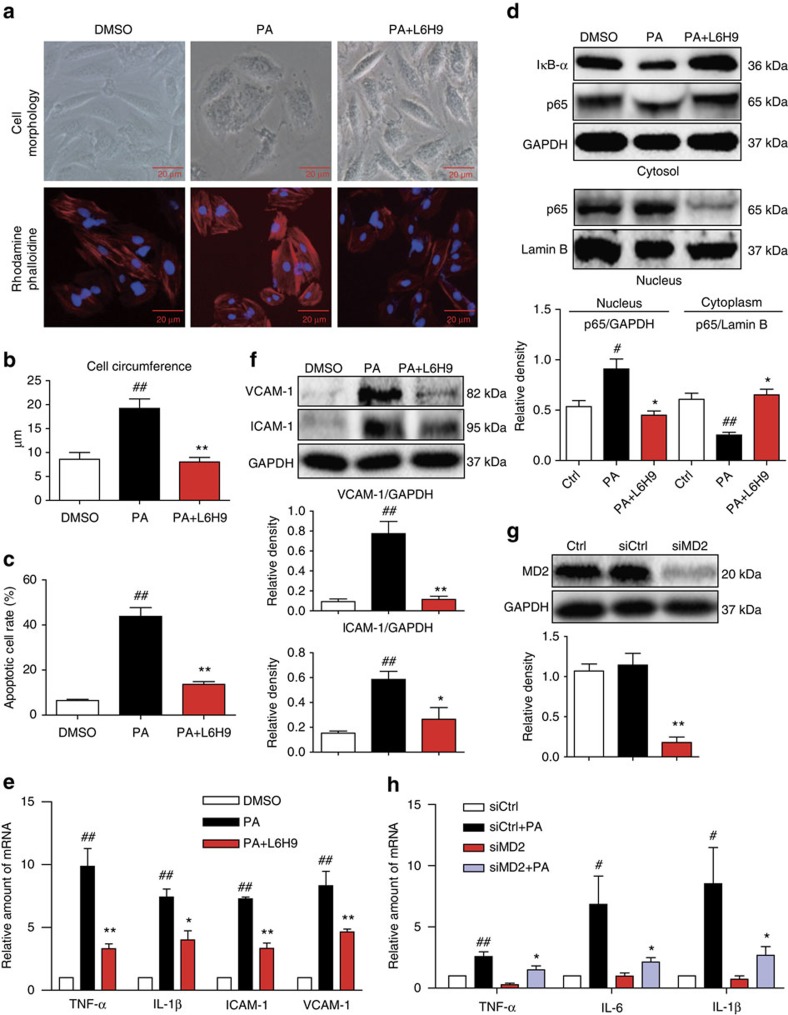
PA induces MD2-dependent inflammatory injury in cardiomyocytes. (**a**,**b**) H9C2 cells were pretreated with 10 μM L6H9 before stimulation with 500 μM PA for 12 h. Representative images of cell morphology captured by phase microscopy (top panel) and F-actin distribution stained by rhodamine–phalloidin (bottom panel) are shown; *n*=3; scale bars=20 μm. Quantification of cell size is shown in **b**. (**c**) Effects of L6H9 pretreatment on apoptosis induced by 500 μM PA for 24 h in H9C2 cells. Quantification of flow cytometric data for apoptotic cells, determined by flow cytometric analysis of FITC-Annexin V and propidium iodide (PI); *n*=3. (**d**) Cytosolic and nuclear protein levels of IκB-α and NF-κB p65 subunit in cells pretreated with L6H9 before stimulation with 500 μM PA for 1 h (representative of *n*=3). Densitometric quantification of NF-κB pathway activation in cells treated with PA is shown below. (**e**) Cytokine expression in H9C2 cells following treatment with 500 μM PA for 6 h. Bar graphs showing relative mRNA values for TNF-α, IL-1β, ICAM-1 and VCAM-1 normalized with GAPDH (*n*=4). (**f**) Representative western blot with densitometric quantification of ICAM-1 and VCAM-1 in cells treated as in **e** (representative of *n*=3). (**g**) Knockdown of *Md2* in H9C2 cells by siRNA transfection. Figure showing protein levels of MD2 in untransfected cells (Ctrl) and in cells transfected with either control/scrambled siRNA (siCtrl) or siRNA targeting *Md2* (siMd2; *n*=4 experiments). (**h**) Effect of *Md2* knockdown on pro-inflammatory gene expression in H9C2 cells stimulated with 500 μM PA for 6 h. Bar graphs showing relative mRNA for TNF-α, IL-6 and IL-1β (GAPDH used as housekeeping gene; *n*=4 experiments). Data in **b**–**h** are reported as mean±s.e.m. and analysed by one-way analysis of varinace, ^#^*P*<0.05, ^##^*P*<0.01, compared with dimethylsulphoxide, Ctrl or siCtrl group; *P<0.05, **P<0.01, compared with PA or siCtrl-PA group.

For the *in vitro* cell studies, we routinely used solutions of PA:BSA ratios of 8:1 to activate cells. At the 8:1 ratio, the amount of free PA is higher than that found in the circulation of obese patients with ratios 4:1 or lower. We evaluated whether lower PA:BSA ratios were also biologically effective in our model system. Comparative studies were made to determine the effects of treatment with PA:BSA ratios of 8:1 or 4:1 on cytokine production in primary rat cardiomyocytes or macrophages. Results indicated that both ratios of PA:BSA significantly induced cytokine production in both cell types (Supplementary Fig. 4a–c) and subsequent injury in primary cardiomyocytes (Supplementary Fig. 4d). We measured cellular stress and indices of inflammatory injury in H9C2 cells pretreated with L6H9 before the exposure of cells to PA (from here on using 8:1 PA:BSA). Results showed hypertrophy in H9C2 cells exposed to PA but not in cells pretreated with MD2 inhibitor L6H9 (Fig. 2a,b). The hypertrophy was accompanied by significant increases in mRNA of hypertrophic markers, atrial and brain natriuretic peptides (ANP and BNP) and myosin heavy chain (MyHC; Supplementary Fig. 5a). Fibrogenic changes manifesting as increased protein levels of collagen 1/4 and TGF-β were also seen in H9C2 cells following treatment with PA. Pretreatment of cells with L6H9 effectively inhibited the PA-increased fibrogenic protein levels (Supplementary Fig. 5a,b). L6H9 also inhibited apoptosis after 24 h of PA treatment as indicated by Annexin V/PI staining (Fig. 2c; Supplementary Fig. 5c). PA induced apoptotic death of H9C2 cells as indicated by decreased Bcl2 and increased Bax protein levels, which were inhibited by MD2 blockade (Supplementary Fig. 5d,e). The findings support an MD2-dependent mechanism by which PA induces inflammatory injury.

The effects of MD2 blockade on PA-stimulated pro-inflammatory gene expression in H9C2 cells were determined next. Nuclear factor-κB (NF-κB) is a downstream target of MD2/TLR4 pathway and regulates the expression of pro-inflammatory genes. MD2 blockade with L6H9 prevented PA-induced inhibitor of κB (IκB-α) degradation and nuclear translocation of the NF-κB p65 (Fig. 2d; Supplementary Fig. 5f). Moreover, L6H9 pretreatment abrogated PA-induced upregulation of key pro-inflammatory genes (TNF-α, IL-1β, ICAM-1 and VCAM-1; Fig. 2e) and their protein expression (Fig. 2f). Furthermore, knockdown of MD2 (Fig. 2g) prevented PA-induced upregulation of pro-inflammatory cytokines (Fig. 2h), consistent with the MD2 inhibition by L6H9. *Md2* silencing also prevented PA-induced cell injury including hypertrophy, fibrogenic factor expression and apoptosis (Supplementary Fig. 6a–c). Key findings from H9C2 cells were confirmed in primary neonatal rat cardiomyocytes. We show that PA at the same concentration used for H9C2 cells, induced primary cardiomyocyte injury manifesting as increased levels of inflammatory molecules, hypertrophy and cell apoptosis (Supplementary Fig. 7a–h). Consistent with our findings in H9C2 cells, MD2 inhibition was effective in preventing the PA-stimulated injury in these primary cardiomyocytes (Supplementary Fig. 7a–h). Since our studies clearly showed increased fibrosis in the heart tissues of mice challenged with PA, we investigated whether PA increases fibrogenic factor expression in cardiac fibroblasts. Although cardiomyocytes are able to synthesize matrix proteins, cardiac fibroblasts are likely the key contributor to this pathologenic event. We found that PA challenge of freshly isolated primary fibroblasts from heart tissues resulted in increased fibrogenic factor expression (Supplementary Fig. 7i). These results provide strong support for a key role of MD2 in mediating PA-induced cardiomyocyte injury.

### PA activates TLR4 signalling through MD2 in macrophages

**Figure 3:**
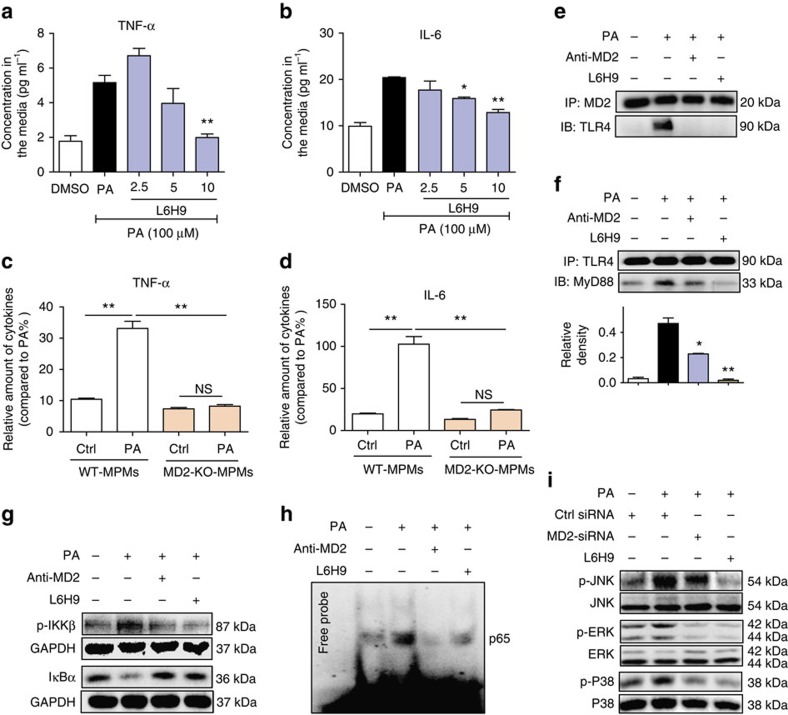
PA induces MD2/TLR4 complex formation and signalling. (**a**,**b**) Effects of MD2 inhibitor L6H9 on secretion of TNF-α (**a**) and IL-6 (**b**) by mouse primary macrophages stimulated with 100 μM PA for 24 h. Cytokine concentrations in the condition medium were measured by ELISA and reported as pg ml^−1^ (*n*=3). (**c**,**d**) Effects of PA stimulation (100 μM for 24 h) on cytokine secretion from primary macrophages isolated from *Md2*^*−\−*^ or wild-type mice, KO-MPM or WT-MPM, respectively. Graphs show relative amounts of TNF-α (**c**) and IL-6 (**d**) (*n*=3). (**e**,**f**) Effects MD2 blockade on formation of the MD2/TLR4–MyD88 complex in macrophages stimulated with 100 μM PA for 5 min. MD2 blockade was achieved by MD2-neutralizing antibody (anti-MD2, 100 ng ml^−1^) or MD2 inhibitor (L6H9, 10 μM). Shown are representative (*n*=3) western blots (IBs) from the co-precipitation (IP) studies for MD2/TLR4 complex formation (**e**) and TLR4/MyD88 complex formation (**f**). (**g**,**h**) Effects of MD2 blockade on NF-κB activity in mouse macrophages stimulated with 100 μM PA (30 min); MD2 blockade was achieved by anti-MD2 or 10 μM L6H9. Shown are (**g**) representative western blot for p-IKKβ and IκB-α (*n*=3), and (**h**) EMSA analysis for NF-κB DNA-binding activity (*n*=3). (**i**) Effects of MD2 inhibition through knockdown or L6H9 pretreatment (10 μM) on MAPK pathway activation in macrophages stimulated with 100 μM PA for 30 min. Shown are representative western blots of phosphorylated JNK, ERK and P38 (densitometric quantification for **g**–**i** from three separate determinations were shown in Supplementary Figs 11a,b and 12a, respectively). Data are reported as mean±s.e.m. and analysed by Student’s *t*-test, *P<0.05, **P<0.01 compared with PA or WT-MPMs; NS, not significant.

We next examined macrophages to determine whether PA mediates pro-inflammatory activity. Macrophages are involved in and contribute to cardiac injury associated with obesity^33^. Numerous studies have also shown that SFAs induce an inflammatory phenotype in macrophages through TLR4 (refs 14, 15, 34). However, our findings indicate that MD2 plays a critical role in the PA activity as *Md2* KO mice failed to show cardiac damage. Therefore, elucidating the role of MD2 in PA-induced macrophage provides novel insight into the pathogenesis of obesity and SFA-associated cardiomyopathy. Freshly isolated mouse primary macrophages (MPMs) were stimulated with PA for 24 h and cytokine secretion was measured by enzyme-linked immunosorbent assay (ELISA). Results indicated that PA stimulated TNF-α and IL-6 release into the culture media, but L6H9 pretreatment abrogated these increases in a dose-dependent manner (Fig. 3a,b). Similarly, L6H9 pretreatment prevented the PA-induced expression of cytokine mRNA (Supplementary Fig. 8a–c). We evaluated MPMs isolated from *Md2*^*−/−*^ mice for further confirmation of the PA effects. Our results show that PA was unable to increase secretion of TNF-α and IL-6 in the *Md2* deficient macrophages (Fig. 3c,d). This was somewhat unexpected since SFAs can activate the TLR2 pathway^34^,^35^,^36^,^37^,^38^ that would not have been targeted in MPMs isolated from the *Md2*^*−/−*^ KO mice. It is possible that PA-mediated TLR2 activation requires much higher concentration of PA than for TLR4. Previous studies reported PA concentrations of 200–750 μM for TLR2 activation, which is >100 μM used in the current study.

We then examined the effect of PA on critical components of the MD2–TLR4 signalling pathway. LPS stimulates the MD2/TLR4 complex to recruit MyD88 to generate pro-inflammatory molecules^27^. Since MyD88 is predominantly linked to FFA-induced inflammatory responses^39^,^40^, we investigated whether PA also stimulates MD2/TLR4 complex formation and MyD88 recruitment in MPMs. We observed that PA stimulated a robust association of MD2 with TLR4, which occurred within 5 min (Fig. 3e), and recruitment of MyD88 to the complex by 1 h (Fig. 3f). However, pretreatment with an MD2-neutralizing antibody (anti-MD2) or L6H9 prevented both MD2/TLR4 complex formation (Fig. 3e) and MyD88 recruitment (Fig. 3f; Supplementary Fig. 9a). An intact MD2/TLR4/MyD88 complex appeared to be essential for PA to induce cytokine production. This is clearly highlighted when the individual proteins in the complex were knocked down with siRNA (Supplementary Fig. 9b–d). One interesting finding of our *in vitro* studies is that a relatively high concentration of PA is required to elicit a MD2-dependent signalling activation as compared with LPS. We performed a dose–response study to determine the minimum concentration of PA needed to activate MD2–TLR4 pathway. Supplementary Figure 10 shows that PA induces TNF-α and IL-6 release at 100 μM or higher in MPMs, possibly indicating this pathway as a chronic response rather than an immediate/rapid response as activated by LPS.

Both NF-κB and mitogen-activated protein kinases (MAPKs) are critical downstream signalling targets of the MD2/TLR4–MyD88 complex. We assessed the effects of PA on these downstream signalling pathways in MPMs. Results indicated that PA activated NF-κB,with increased phosphorylation of IκB kinase (IKKβ), IκB-α degradation and NF-κB DNA binding (Fig. 3g,h; densitometry shown in Supplementary Fig. 11a,b). Blocking MD2 with anti-MD2 or L6H9 prevented the PA-stimulated IκB-α degradation, IKKβ phosphorylation (Fig. 3g; Supplementary Fig. 11c), NF-κB DNA binding (Fig. 3h) and nuclear translocation of the NF-κB p65 subunit (Supplementary Fig. 11d,e). PA also activated the MAPK pathway, with increased phosphorylation of JNK, ERK and P38, whereas MD2 blockade by siRNA knockdown, L6H9 pretreatment or anti-MD2, inhibited the PA-induced phosphorylation of MAPKs (Fig. 3i; Supplementary Fig. 12a–h). The activation of these two pathways in mediating PA-induced cytokine expression was confirmed with pharmacological inhibitors of MAPKs and NF-κB (Supplementary Fig. 12i–k). The findings indicated that like LPS, PA stimulated the formation of MD2/TLR4–MyD88 complex, resulting in activation of MAPKs/NF-κB signalling for the regulation of pro-inflammatory molecules.

### Evidence for direct PA interactions with MD2

There is substantial evidence for a functionally important relationship between SFAs and TLR4 signalling, but the molecular mechanisms remain unclear. SFAs are known to induce TLR4-depedent gene expression by promoting dimerization of TLR4 in macrophages^41^. Our *in vivo* and *in vitro* studies provided evidence that MD2 was crucial in the PA-induced inflammatory injury, implicating MD2–TLR4 involvement. We used a combination of cell-free and cell-based assays to test the idea that PA directly interacted with MD2 in the activation of MD2/TLR4–MyD88 signalling pathway.

**Figure 4:**
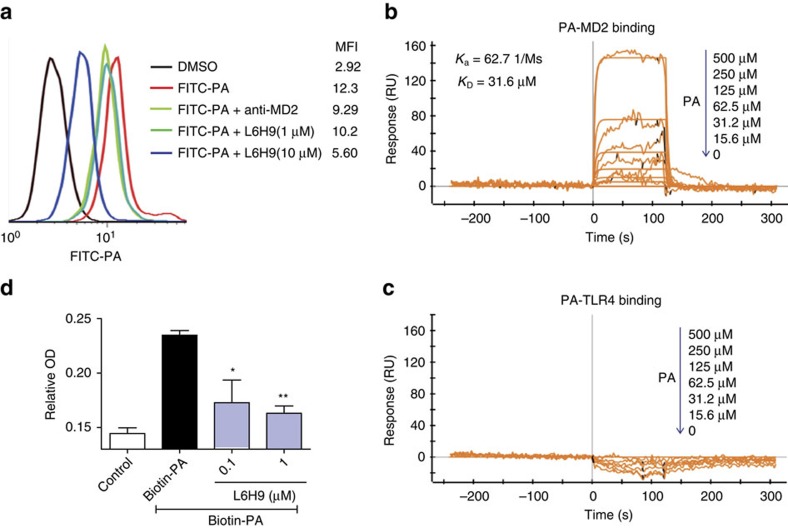
PA interacts directly with MD2 to activate TLR4 signalling. (**a**) Flow cytometric analysis showing the effects of MD2 blockade on the binding of FITC-PA (50 μM) to the cell surface of mouse macrophages. MD2 was blocked with either anti-MD2 (100 ng ml^−1^) or L6H9 (1 and 10 μM). (**b**,**c**) Representative graphs from surface plasmon resonance spectroscopy analysis showing the binding of increasing concentrations of PA with human recombinant MD2 (**b**) or TLR4 (**c**). The *K*_a_ and *K*_D_ values are indicated on the upper left. (**d**) Binding of biotin-PA with rhMD2 pre-absorbed in the ELISA plate (OD values reported as mean±s.e.m. and analysed by Student’s *t*-test; *P<0.05, **P<0.01, compared with biotin-PA group). *n*=4 for all *in vitro* experiments.

We first evaluated the ability of fluorescein isothiocyanate (FITC)-conjugated PA (FITC-PA) to bind to macrophage cell surface. MPMs incubated with FITC-PA showed a significantly increased mean fluorescence intensity compared with control (Fig. 4a). Interestingly, MD2 blockade with anti-MD2 or L6H9 reduced FITC-PA binding to MPM cell surface (Fig. 4a), implicating MD2 requirement for PA cell surface binding. We next used surface plasmon resonance (SPR) to evaluate direct interactions between the binding partners. Results indicated that PA interacted directly with recombinant human MD2 (rhMD2) with a *K*_D_ of 31.6 μM using a ProteOn XPR36 Protein Interaction Assay system (Fig. 4b). However, no direct interaction occurred between PA and TLR4 (Fig. 4c). These findings were further corroborated using an ELISA-based assay that detected binding of biotinylated PA (biotin-PA) with immobilized rhMD2 (Fig. 4d). However, the co-incubation with L6H9 prevented >50% of biotin-PA binding to rhMD2, suggesting competitive interaction with PA for binding to rhMD2 (Fig. 4d). Together, the results obtained from the cell-free and cell-based studies indicated that PA directly interacted with MD2, a new finding that suggests a potential mechanism for activating MD2/TLR4 signalling.

Molecular docking simulations based on the multiple ligand simultaneous docking (MLSD) program^42^ was used to predict binding poses of PA within the binding pocket of MD2 and the docking scores (that is, free energy of binding; Methods in Supplemental Section), which included analysis of the crystal structure of human MD2 in complex with the 14-carbon myristic acid (MA; PDB ID 2E56) as control^43^. The simulation results in Supplementary Figs 13 and 14, indicated that the binding of three molecules of PA (or MA) presented with the lowest docking scores (that is, high binding affinity) and appropriate positioning of the fatty acid within the MD2 cavity. Moreover, molecular dynamic (MD) simulations of the binding poses indicated that the MD2/PA (1:3) and MD2/MA (1:3) structures showed similar structural stability, with root mean square deviation values of ∼2.5 Å (Supplementary Fig. 15). The molecular simulation results indicated that likely three molecules of PA can bind within the MD2 pocket, creating a stable complex of relatively high affinity.

Collectively, our findings show that PA bound MD2 and activated the TLR4 signalling pathway. Recently, Fetuin A was reported to act as an adaptor protein in linking PA to TLR4 signalling^44^. To investigate whether Fetuin A was involved in our platform, we assessed the effect of exogenously supplied Fetuin A on PA-induced cytokine expression in primary macrophages. PA was also able to cause complex formation between MD2-Fetuin A-TLR4 and induce IL-6 and TNF-α production (Supplementary Fig. 16a–d). However, this complex formation was not evident in macrophages isolated from *Md*2 KO mice (Supplementary Fig. 16a). To remove the effect of Fetuin A present in the serum, macrophages were cultured in serum-free media and exposed to exogenous Fetuin A. We show that Fetuin A was able to cooperate with MD2 to increase the secretion of cytokines (Supplementary Fig. 16c,d). These results confirmed the previous report and indicated that Fetuin A may cooperate in the signalling pathway; however, MD2 is the critical protein.

Our findings have also raised an interesting question as to whether MD2 binding with fatty acids was specific to SFAs. To explore this, we assessed the ability of unsaturated oleic and linoleic acids to bind MD2 and induce an inflammatory phenotype. Our SPR results from ForteBio Octet HTX system not only confirmed the interaction of PA-rhMD2 (*K*_D_=95 μM), but also showed that the unsaturated fat acids, oleic and linoleic acids, bound to rhMD2 with *K*_D_ values of 35 and 54 μM, respectively (Supplementary Fig. 17a–c). However, both oleic and linoleic acids failed to activate MD2–TLR4 complex formation at the same effective concentration as PA (Supplementary Fig. 18a,b) or induce expression of pro-inflammatory cytokines (Supplementary Fig. 18c–f) in macrophages or H9C2 cells. Furthermore, when used at the same concentration as PA, oleic acid failed to induce cardiac inflammatory injury in mice (Supplementary Figs 19 and 20). These findings indicated that MD2 was able to bind unsaturated fatty acids but mediated pro-inflammatory activity only in response to SFAs.

### *Md*2 knockout reduces myocardial inflammatory injury in HFD

**Figure 5:**
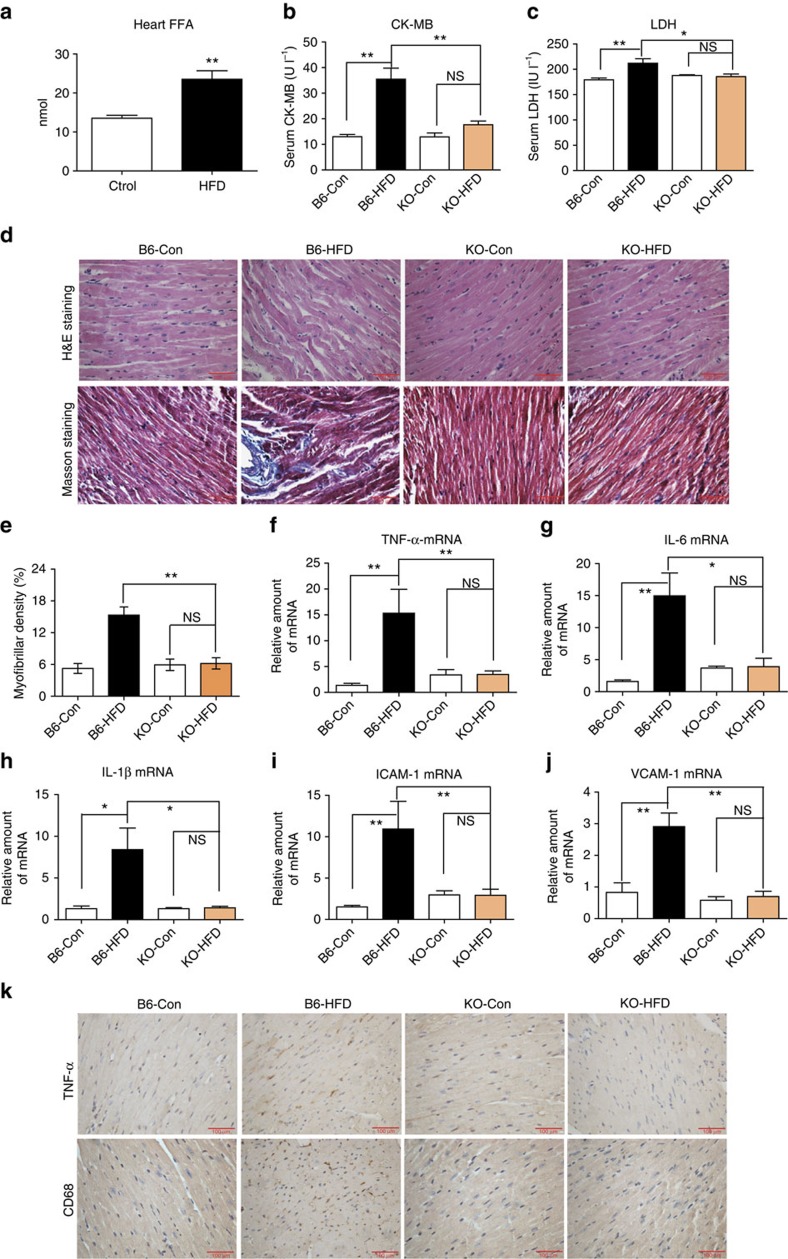
*Md2* knockout reduces myocardial injury in HFD. Wild-type (B6) and *Md2*^*−/−*^ (KO) mice were fed a HFD for 4 months, and blood and heart tissue collected for evaluation of myocardial inflammatory injury. (**a**) Levels of FFA in 20mg heart tissue of each mouse fed a control (Ctrol) diet or HFD; FFA reported as mean ± s.e.m. and analysed by Student’s *t*-test, **P<0.01 compared with Ctrol diet; *n*=8). (**b**,**c**) Serum markers of cell injury indicated by CK-MB and LDH (*n*=8). (**d**) Upper panel shows representative micrographs of heart tissue stained with hematoxylin and eosin; lower panel shows histochemical staining for connective tissue using Masson’s trichrome; *n*=4; scale bars, 100 μm. (**e**) Quantification of fibrosis indicated by trichrome staining, *n*=3. (**f**–**j**) RT–qPCR determination of inflammatory genes and adhesion molecules in heart tissue (*n*=4). Figure showing TNF-α (**f**), IL-6 (**g**), IL-1β (**h**), ICAM-1 (**i**) and VCAM-1 (**j**). mRNA was normalized to housekeeping gene β-actin, and reported relative to B6-Con. (**k**) Representative immunohistochemical analysis of TNF-α and CD68 (immunoreactivity=brown); *n*=4; scale bars, 100 μm. Data in **b**,**c** and **e**–**j** are reported as mean±s.e.m. and analysed by Student’s *t*-test; *P<0.05, **P<0.01, B6-HFD group compared with control (Con) or KO groups; NS, not significant.

Much evidence indicates that the elevated FFAs levels in obese subjects^7^,^8^ are linked to increased cardiovascular mortality^45^. Therefore, we investigated the role of MD2 in mediating cardiac damage in a clinically relevant model of obesity. Since we previously reported that HFD-fed mice develop myocardial inflammatory injury^19^, we determined whether MD2 blockade suppresses this HFD-induced myocardial injury. Wild-type (B6) and *Md2*^*−/−*^ (KO) mice were fed a HFD or control rodent diet for 4 months. HFD resulted in dyslipidemia in both the B6 and KO mice, as evidenced by elevated circulating levels of low-density lipoproteins (LDLs), triglycerides (TG) and total cholesterol (TC; Supplementary Fig. 21a–c), confirming our previous findings^19^. HFD also increased local FFA level in mouse heart (Fig. 5a). In addition, serum levels of CK-MB and LDH were elevated (Fig. 5b,c). Histological analysis of cardiac tissues from the B6-HFD mice showed disorganized myofibers and increased collagen deposition as detected by Masson’s trichrome (Fig. 5d,e) and Sirius red staining (Supplementary Fig. 21d), which were consistent with increased mRNA levels of pro-fibrotic genes (Supplementary Fig. 21e–g). Evaluation of the *Md2* KO-HFD indicated lack of cardiac myofiber disorganization and fibrotic changes (Fig. 5b–e; Supplementary Fig. 21d–g), as well as lack of alteration in CK-MB (Fig. 5b) and LDH (Fig. 5c).

In addition, HFD significantly increased expression of hypertrophic indicators, ANP, BNP and MYH in B6 mice, indicative of increased cardiac stress. However, the levels of these markers remained similar to the normal diet controls and the KO-HFD mice (Supplementary Fig. 21h–j). Echocardiographic assessment of cardiac function showed significant cardiac dysfunction in the B6-HFD mice but not the KO-HFD mice (Table 1). Although we observed the left ventricle ejection fraction of 67% in mice maintained on HFD that is not considered low according to a few published reports^46^, it should be noted that compared with mice on control diet, these fraction values were significantly depressed. Together with data showing structural alterations, our functional parameters were indicative of significant cardiac functional deficits. These findings support the notion that MD2 is essential for the development/progression of obesity-associated cardiac tissue injury and dysfunction.

We determined the effects of *Md2* KO on cardiac tissue levels of inflammatory genes. The quantitative PCR (qPCR) analysis indicated that HFD upregulated pro-inflammatory cytokines (TNF-α, IL-6 and IL-1β) and adhesion molecules (ICAM-1 and VCAM-1) in the B6 mice, but not the KO mice (Fig. 5f–j). Immunostaining also revealed decreased TNF-α levels in KO-HFD mice compared with B6-HFD mice (Fig. 5k; Supplementary Fig. 22a). The increased histochemical detection of CD68, expressed predominantly by macrophages, in cardiac tissue of the B6-HFD mice further corroborated the increased ICAM-1 and VCAM-1 expression levels (Fig. 5k; Supplementary Fig. 22b). The findings provide strong evidence for the potential contribution of MD2 in obesity-related myocardial inflammatory injury.

## Discussion

Excessive lipids activate detrimental signalling pathways leading to the generation of inflammatory molecules and tissue remodelling, resulting in cardiomyopathy^14^,^47^. Identification of key signalling molecules underlying cardiac inflammation may lead to novel treatments for obesity-associated complications. Our findings provide strong evidence that MD2 is an important accessory protein for SFAs in inducing pro-inflammatory activities, and myocardial injury and dysfunction. The two key results are as follows: (1) studies of cell surface binding and cell-free protein–protein interactions indicated that PA directly bound to MD2, supporting a mechanism of direct PA (and possibly other SFAs) binding to MD2 to activate TLR4 signalling, and (2) Blockade of MD2 prevented production of pro-inflammatory molecules in myocardial tissue, cardiac tissue remodelling and cardiac dysfunction in mice with hyperlipidemia and/or obesity. In addition, both cardiomyocytes and macrophages acquired an MD2-dependent pro-inflammatory phenotype when challenged with PA. Our results underscore MD2 as a central and necessary protein in SFA-mediated myocardial inflammatory injury.

The direct relationship between SFAs and MD2 in myocardial inflammatory injury is a new observation in the field. Our studies revealed a molecular mechanism by which SFAs activated TLR4/MD2-dependent signal transduction. Although SFAs are known to regulate pro-inflammatory activities through TLR4 (refs 14, 15, 16, 22, 24, 25), the precise interactions and mechanisms have remained elusive. A recent study assessed binding of ^14^C-labelled stearic and oleic acids to a complex of FLAG-tagged extracellular TLR4 fused to full-length MD2 (ref. 16). The study concluded that fatty acids do not directly bind to TLR4/MD2. However, the binding assay with ^14^C-labelled fatty acids alone is insufficient to rule out direct binding of fatty acids to the TLR4/MD2 complex. Using multiple approaches, we present strong evidence here that PA directly bound MD2 and induced MD2/TLR4 complex formation and production of pro-inflammatory cytokines. The structural analyses using recombinant proteins coupled with molecular simulations supported a mechanism of direct binding, at least by PA, within the deep pocket of MD2. This binding initiated complex formation with TLR4, recruitment of MyD88 and subsequent transduction of signalling pathways. The molecular simulation results (using both MLSD and RMSD methods described in the Supplemental Section) provided further support of direct PA–MD2 binding and predicted three molecules of PA binding within the MD2 cavity. Moreover, this appear to produce a relatively stable complex, with appropriate positioning of fatty acids and reasonable binding affinity. The generation of a crystal structure for the PA–MD2 complex, though has been difficult, is much needed for confirmation of this important finding.

In our study, the concentration of PA needed to elicit MD2–TLR4 signalling was relatively high compared with what is known about LPS. Although the binding was specific, the high PA levels required to activate TLR4 may be attributed to several reasons. It is possible that LPS produces rapid responses whereas SFAs were involved in more chronic insults consistent with low-grade inflammation. There may be co-proteins involved in the rapid LPS responsiveness. Studies have shown that CD14 is one of these co-proteins that allow low-level LPS to activate TLR4 (refs 48, 49). Nonetheless, our evidence of direct binding of PA to MD2 paved the way for future detailed investigations into PA responsiveness and TLR4 signalling.

The HFD-associated myocardial inflammatory injury was likely attributed to both direct and indirect effects of PA and/or other SFAs on cardiac cells. We observed that cardiomyocytes were susceptible to direct PA stimulation, resulting in altered morphology, cellular injury and inflammatory responses. As in other tissues including adipose tissue^50^ and pancreas^23^, cardiomyocytes are exposed to indirect insults within the highly pro-inflammatory environment created by PA. We found increased CD68 immunoreactivity and expression of adhesion molecules in heart tissues of PA-challenged or HFD-fed mice, indicative of increased macrophage recruitment. We also found that PA-stimulated macrophages presented an inflammatory phenotype which was also dependent on MD2. However, the exact contribution of the cardiac cells in the inflammatory injury we observed remains to be determined. The heart injury present in the PA or HFD mouse models was likely from a combination of direct PA effects on cardiomyocytes and effects secondary to activated leukocytes in the local tissue environment. In addition, fibroblasts may be involved in PA-induced cardiac injury as can be safely assumed based on the observed cardiac fibrosis. Supplementary Fig. 7i showed that PA stimulation could increase TGF-β and collagen-4 expression in rat primary cardiac fibroblasts. It is unclear which cell type(s) within the local myocardial tissue environment are predominant factors driving the tissue injury and remodelling.

Several pieces of evidence, including findings from this study, indicate that the ability PA (or SFAs) to activate signalling was likely attributed to its SFA state. The removal of SFAs or a change to unsaturated fatty acids on the Lipid A moiety of LPS results in a complete loss of endotoxic activity^14^. We found that despite the binding of unsaturated fatty acids to MD2, they did not induce MD2–TLR4 complex formation nor activate downstream TLR4 signalling. These unsaturated fatty acids were unable to induce cardiac inflammatory phenotype both *in vitro* and *in vivo*. Thus, likely, unsaturated FAs were not significant contributors to myocardial remodelling and injury in obesity. Furthermore, studies by Lee *et al*.^51^ and Hwang *et al*.^52^ found that the SFAs activated TLR4 signalling, but polyunsaturated FAs, particularly docosahexaenoic acid, inhibited the activation by FFAs or LPS, indicating a reciprocal modulation of TLR4 activation between the two classes of FAs. Our observation that unsaturated FAs bound MD2 suggests an intriguing possibility that unsaturated FAs may competitively inhibit SFAs to modulate TLR4 signalling response and chronic inflammation. Such reciprocal regulation of TLR4 by SFAs and unsaturated FAs could function as a lipid sensor in the cardiovascular system. This is certainly a focus of future studies.

In summary, our findings indicated that the PA- and HFD-induced myocardial injury was dependent on MD2, the dependency of which was likely regulated by direct binding with PA. The findings are new mechanistic insights linking FFAs in obesity and immunity in cardiovascular diseases.

## Methods

### Materials and reagents

PA was obtained from Sigma-Aldrich (St Louis, MO). Biotin-PA (catalogue no.: LP14590 at 98.0% purity) and FITC-PA (catalogue no.: LP14590-03 at 96.7% purity) were purchased from Shanghai Leon Chemical Ltd (Shanghai, China). One Step TUNEL Apoptosis Assay kit, Rhodamine phalloidin staining kit, enhanced chemiluminescence reagent, FITC-annexin V apoptosis detection kit, electrophoretic mobility shift assay (EMSA) kit, NF-κB consensus oligonucleotides, BAY 11-7082, SB203580, PD98059, SP600125 and Protein G Agarose beads were purchased from Beyotime Biotech (Beijing, China, MD2-neutralizing antibody (anti-MD2) was obtained from R&D Systems. The following antibodies with their working dilutions indicated in parenthesis were purchased from Santa Cruz Technology (Santa Cruz, CA): MD2 (1:200), TLR4 (1:200), MyD88 (1:200), IκBα (1:200), p-IKKβ (1:200), NF-κB p65 subunit (1:200), Cleaved-PARP (1:200), GAPDH (1:200), Bax (1:200), Bcl2 (1:200), TGF-β (1:200), collagen-4 (1:200), CD68 (1:200), VCAM-1 (1:200), ICAM-1 (1:200), Lamin B (1:200), and HRP- and PE-conjugated secondary antibodies (1:3,000). Antibodies against TNF-α were purchased from Abcam (Cambridge, MA). Antibodies to p-JNK (1:1,000), JNK (1:1,000), p-ERK (1:200), ERK (1:200), p-P38 (1:1,000), P38 (1:1,000) and cleaved caspase-3 (1:1,000) were purchased from Cell Signaling Technology (Danvers, MA).

ELISA kits for mouse TNF-α, IL-6 or IL-1β were purchased from eBioscence (San Diego, CA). Nuclear and Cytoplasmic Protein Extraction kits were from KeyGEN Biotech (Nanjing, China). Polyvinylidene difluoride membrane was purchased from Bio-Rad Laboratory (Hercules, CA). M-MLV Platinum RT–qPCR kit and TRIZOL were purchased from Invitrogen (Carlsbad, CA). siRNA sequences were synthesized (Supplementary Table 1) by GenePharma (Shanghai, China).

The small-molecule MD2 inhibitor, L6H9, was synthesized by our group and prepared with a purity of 99.2% for studies^32^. For *in vitro* experiments, L6H9 was dissolved in dimethylsulphoxide; for *in vivo*, L6H9 was dissolved in CMC-Na buffer (0.5%).

### Animal studies

All animal care and experimental procedures complied with the ‘The Detailed Rules and Regulations of Medical Animal Experiments Administration and Implementation’ (Document no. 1998-55, Ministry of Public Health, China), and were approved by the Wenzhou Medical University Animal Policy and Welfare Committee.

Male 8-week-old C57BL/6 mice weighing 18–22 g were obtained from the Wenzhou Medical University Animal Center (Zhejiang, China). Age-matched male *Md2*^*–/–*^ mice (B6.129P2-Ly96 KO) on C57BL/6 background were provided by Riken BioResource Center of Japan (Tsukuba, Ibaraki, Japan). The mice were housed in an environmentally controlled room at 22±2.0 °C and 50±5% humidity with a 12-h:12-h light/dark cycle, and given food and water *ad libitum*.

For the PA mouse model, wild-type C57BL/6 (B6) and *Md2*^*−/−*^ (KO) mice were injected with 500 μl solution of 5 mM PA (via tail vein), NS control or BSA vehicle control twice daily for 7 days (*n*=8 per group). For studies involving L6H9 inhibitor, mice were administered 20 mg kg^−1^ per day L6H9 (dissolved in 100 mM citrate buffer (pH 4.5)) 3 days before PA challenge. Mice were killed, and blood and heart tissues were collected at the end of the study. Blood samples were used to prepare serum for the measurement of TC, LDL and TG levels using commercial kits. Excised heart tissues were weighed and fixed in 4% paraformaldehyde for histological analysis or frozen in liquid nitrogen for other analyses including gene and protein level determinations.

The role of MD2 was also investigated in a mouse model of HFD-induced obesity. Wild-type and *Md2*^*–/–*^ mice were acclimated for 2 weeks before starting the diet regimen. Mice were fed a HFD containing 60 kcal.% fat, 20 kcal.% protein and 20 kcal.% carbohydrate (catalogue no. MD12033; MediScience Diets Co. Ltd, Yangzhou, China) or a standard chow containing 10 kcal.% fat, 20 kcal.% protein and 70 kcal.% carbohydrate (catalogue no. MD12031; *n*=8 per group). Body weights were recorded weekly. After 4 months, mice were killed and blood samples were collected for serum preparation. Heart tissues were excised, weight and processed as described for the PA challenge mouse model.

### Cell isolation and culture

The embryonic rat heart-derived cell line H9C2 was obtained from the Shanghai Institute of Biochemistry and Cell Biology (Shanghai, China) and cultured in DMEM medium (Gibco, Eggenstein, Germany) containing 5.5 mmol l^−1^ of D-glucose supplemented with 10% fetal bovine serum (FBS), 100 U ml^−1^ of penicillin and 100 mg ml^−1^ of streptomycin.

Primary macrophages from B6 and *Md2* KO mice were isolated and cultured as follows^53^. Mice were stimulated by intraperitoneal injection with 2 ml of 6% thioglycollate solution (0.3 g beef extract, 1.0 g tryptone and 0.5 g NaCl dissolved in 100 ml double-distilled H_2_O and filtered through 0.22-μm filter) and kept in a pathogen-free environment for 2 days. Macrophages were collected by washing the peritoneal cavity with 8 ml of PBS containing 30 mM of EDTA, centrifuged and resuspended in RPMI-1640 medium (Gibco/BRL life Technologies, Eggenstein, Germany) with 10% FBS, 100 U ml^−1^ penicillin and 100 mg ml^−1^ streptomycin. Non-adherent cells were removed by washing with medium 3 h after seeding, and firmly adherent MPMs on culture dishes were used for experiments.

Neonatal rat primary cardiomyocytes and fibroblasts were isolated and cultured based on the modified method by Laugwitz *et al*.^54^ and Guo *et al*.^55^ For cell isolation, rat hearts were cut into pieces, washed repetitively in ice-cold HBSS without Ca^2+^, predigested overnight in 0.5 mg ml^−1^ trypsin–HBSS solution at 4 °C under constant shaking, followed by four rounds of 10 min digestions with 240 U ml^−1^ collagenase type II in HBSS at 37 °C. The digestate was filtered through gauze to remove tissue debris, centrifuged, exposed to serial Ca^2+^ concentrations up to 900 μM, plated onto laminin-coated culture dishes and replaced with serum-free Eagle’s MEM with 0.1% BSA to allow attachment of cardiomyocytes. The collected buffer from the above step, containing cardiofibroblasts, was centrifuged, resuspended in DMEM with 10% FBS, and plated onto culture dishes for study.

### Fatty acid–albumin complex

PA was solubilized in 0.1 N sodium hydroxide and combined with fatty acid-free and low-endotoxin BSA (5%). The albumin levels in the 5 mM PA solution was 627 μM. Fatty acid–albumin complex solution was freshly prepared before each experiment.

### Apoptosis assays

Tissue sections of 5-μm thickness were used for the TUNEL apoptosis detection using One Step TUNEL Apoptosis Assay kit (Beyotime Biotech, Beijing, China). For cells, apoptosis was detected by Annexin V and propidium iodide staining.

### siRNA transfections

Cells were transfected with 50 pmol ml^−1^ of siRNA against MD2, TLR4, MyD88 or negative control (ctrl; Supplementary Table 1; GenePharma, Shanghai, China) using Lipofectamine2000 transfection reagent for 24 h. Cells were then washed and cultured in fresh complete medium. Gene knockdown was confirmed by qPCR and western blot analysis.

### Real-time qPCR

Total RNA was isolated from cells or tissues using TRIZOL reagent. Reverse transcription and qPCR (RT–qPCR) were performed using M-MLV Platinum RT–qPCR kit in Eppendorf Real Plex 4 instrument (Eppendorf, Hamburg, Germany). Primers for genes including TNF-α, IL-6, ICAM-1, MCP-1, collagen 1, TGF-β, ANP, BNP and β-actin were obtained from Invitrogen (Shanghai, China). The primer sequences for all genes used are shown in Supplementary Table 2. The relative amount of each gene was normalized to the amount of β-actin.

### Histology and immunohistochemistry

Heart specimens were fixed in 10% neutral-buffered formalin and embedded in paraffin. Tissue sections were subjected to hematoxylin and eosin staining for routine histological analysis of myofibers. Paraffin sections were also stained with 0.1% Sirius Red F3B and 1.3% saturated aqueous solution of picric acid to evaluate type IV collagen deposition. In addition, sections were stained with Masson’s trichrome stain for connective tissue using routine methods.

Immunohistochemistry for CD68 and TNF-α was performed on dewaxed and rehydrated sections, which were incubated with the primary antibodies at respective working dilutions of 1:50 and 1:500 for overnight at 4 °C. Secondary horseradish peroxidase-conjugated secondary antibodies and DAB were used for detection. Sections were counterstained with hematoxylin to visualize nuclei. All images were taken using bright-field illumination on an epifluorescence microscope equipped with digital camera (Nikon, Japan).

### Cell immunofluorescence staining

For morphology assessment, the cells were fixed with 4% paraformaldehyde, permeabilized with 0.1% Triton X-100, and stained with rhodamine phalloidin at a concentration of 50 μg ml^−1^ for 30 min at room temperature for fluorescence microscopy. For immunofluorescent detection of NF-κB p65, cells were fixed with 4% paraformaldehyde and permeabilized with 100% methanol at −20 °C for 5 min. Cells were then washed and incubated with primary antibodies for NF-κB p65 subunit (1:200) overnight at 4 °C. Phycoerythrin-conjugated secondary antibody was used for detection. Cells were counterstained with 4,6-diamidino-2-phenylindole.

### Western blot analysis and immunoprecipitation

Cell and tissue lysate homogenates were resolved in 10% SDS-acrylamide gels by electrophoresis, and transferred to polyvinylidene difluoride membrane. Membranes were then probed with specific primary and secondary antibodies. The immune complexes were visualized by exposure in a ChemiDoc XRS+ system (Bio-Rad, Hercules, CA) after adding enhanced chemiluminescence reagent. For immunoprecipitation, cell extracts were prepared and incubated with anti-MD2 (or anti-TLR4) antibody (1:200) for 1 h, and immunoprecipitation was made with protein G-sepharose beads at 4 °C overnight. Samples were immunoblotted for detection of TLR4 (or MyD88) as co-precipitated protein. Band densities were quantified using Image J software (NIH, Bethesda, MD). Uncropped western blots are shown in Supplementary Fig. 23.

### Electrophoretic mobility shift assay

Nuclear extracts from cells were prepared using the Nuclear and Cytoplasmic Protein Extraction kit and protein concentrations were determined. NF-κB activity was determined using EMSA kit as described previously^56^. In brief, nuclear protein-oligonucleotide reactions were run on a non-denaturing, 4% acrylamide (29:1 acrylamide to bisacrylamide) gel. After electrophoresis, DNA–protein complex was transferred to a nylon membrane and cross-linked. The biotinylated-labelled DNA was detected by chemiluminescence. The NF-κB consensus oligonucleotides (double-stranded DNA) were: 5′-AGTTGAGGGGACTTTCCCAGGC-3′.

### Enzyme-linked immunosorbent assay for cytokine detection

Serum, conditioned medium, and myocardial tissue homogenates were used for the detected for cytokines. TNF-α, IL-6 and IL-1β levels were measured by ELISA kits (eBioscence, Inc., San Diego, CA). Total amount of proteins was calculated using the standard curve method.

### Serum markers and lipids of cardiac damage

The components of serum lipid including the total TG, LDL, TC, CK-MB and LDH were determined using commercial assay kits (Nanjing Jiancheng BioTech. Co. Ltd, Jiangsu, China).

### Determination of cardiac FFA level

FFA level in myocardial tissue homogenates was determined by a commercial ELISA kit (Nanjing Jiancheng BioTech. Co. Ltd, Jiangsu, China).

### Detection of PA interactions with MD2

The interaction between PA and MD2 was examined using cell-based and cell-free approaches. We first detected cell surface binding of PA by flow cytometry using techniques described previously^57^. In brief, FITC-labelled PA (FITC-PA) or LPS (FITC-LPS) was added to primary macrophages and cells were cultured for 1 h in the presence or absence of L6H9. After washing, cells bound with FITC-PA or FITC-LPS were detected by flow cytometry.

The direct binding of PA or LPS with rhMD2 was determined by cell-free ELISA. rhMD2 protein (4 μg ml^−1^) was immobilized to ELISA microplate surface, biotin-labelled PA or LPS added in 10 mM Tris-HCl (pH 7), and incubated for 2 h in the presence or absence of L6H9. After rinsing, horseradish peroxidase-labelled streptavidin was added, reacted with o-phenylenediamine (2 mg ml^−1^) containing 0.15% H_2_O_2_ for 20 min, stopped with 1 N H_2_SO_4_, and absorbance read at 490 nm.

In addition, the binding affinity of PA or L6H9 to rhMD2, mutated rhMD2, or rhTLR4 was determined by SPR spectroscopy using a ProteOn XPR36 Protein Interaction Assay system (Bio-Rad Laboratories, Hercules, CA) with a HTE sensor chip (ProteOn, #176-5033) or a ForteBio Octet HTX system (Pall Corporation, Menlo Park, CA). In ProteOn system, in brief, the recombinant protein (in acetic acid buffer, pH 5.5) was loaded onto the sensors and 10 mM NiSO4 was added for activation of the chip. PA or L6H9 samples were prepared with running buffer (PBS, 0.1% SDS, 5% dimethylsulphoxide). Sensor and sample plates were placed on the instrument, and interactions determined according to manufacturer’s instruction at a flow rate of 30 μl min^−1^ for 120 s during the association phase followed by 120 s for the dissociation phase at 25 °C. The data were analysed with ProteOn manager software and binding kinetic parameters including the *K*_D_ values were calculated by global fitting of the kinetic data using a 1:1 Langmuir binding model.

Fluorescence measurements of 1,1′-bis(anilino)-4,4’-bis(naphthalene)-8,8′-disulfonate displacement were performed with a spectraMax M5 (Molecular Devices, Sunnyvale, CA). All measurements were done at 25 °C in a 1-cm path-length quartz cuvette. In brief, 1,1′-bis(anilino)-4,4’-bis(naphthalene)-8,8′-disulfonate (5 μM) and rhMD2 protein (5 nM) were mixed in PBS (pH 7.4) and incubated to reach stable relative fluorescence units emitted at 430–590 nm under excitation at 385 nm. Non-fluorescent L6H9 at indicated concentrations was then treated for 5 min, and followed by measuring relative fluorescence units emitted at 430–590 nm.

### Molecular simulation analyses of PA–MD2 interactions

Molecular docking analysis to predict binding poses of PA in the active site of MD2 was made using the MLSD program^42^. To date, only the crystal structure of human MD2 in complex with the 14-carbon MA (PDB ID 2E56)^43^ is available, but not with PA; therefore, our simulaton analyses included MA-MD2 as control. The structure of human MD2 was obtained by removing three MA molecules from the co-crystal structure of human MD2 and MA. A grid box size of 60 × 60 × 40 dimensions with a spacing of 0.375 Å between the grid points was implemented and covered almost the entire MD2-binding site. Lamarckian genetic algorithm and particle swarm optimization were used as a searching method depending on the dimensionality of search space. Particle swarm optimization, a searching method inspired by bird flocking, was used for MLSD with relatively high dimensions of conformational space. The docking parameters were as follows: trials of 200 dockings, the number of individuals in population was set to 300, maximum number of generations was 50,000 and other settings were set default. AutoDockTools and PyMol was used to analyse the docking results^58^,^59^.

MD simulations were used to verify the binding poses of PA in the active site of MD2. Furthermore, apo-MD2 (with removal of MA from the MD2 complex, PDB ID 2E56) and the crystal structure MD2 with MA (PDB ID 2E56) were used as control^43^. The charges of PA and MA were first obtained by the restrained electrostatic potential fitting technique based on the electrostatic potential computed by Gaussian 09 at Hartree–Fock SCF/6-31G* level of theory^60^. Afterwards, the topology and parameter files of PA and MA were generated by using the antechamber module in AMBER11 simulation package^61^. Molecular mechanics parameters from the ff99SB and GAFF force fields were assigned to the protein and the ligands, respectively, using the LEaP module of AMBER11 software packages^62^,^63^. These systems were all solvated in a box of TIP3P water molecules with a hydration shell of 10 Å. In addition, an appropriate number of chloride ions were used to neutralize these systems.

MD simulations were carried out with AMBER11 (Assisted Model Building with Energy Refinement 11) software packages and three systems using the same protocol^64^,^65^. Before the MD productive simulation, we performed an equilibration protocol consisting of an initial minimization of the water box of 5,000 steps, 2,500 steps for the steepest descent and 2,500 steps in the conjugate gradient. The TIP3P water box was heated at constant volume until 300 K using a time constant for the heat bath with a coupling time of 100 ps. Equilibration was at 300 K and constant pressure of 100 ps. Before the production of MD, 100 ps for the whole system was equilibrated at a constant pressure of 1 bar. Finally, 30 ns of MD simulations without any restraints were performed. Furthermore, non-bonded interactions were cut off at 8.0 Å. Periodic boundary conditions were turned on in every step of the whole process^66^. Particle mesh Ewald was also performed to deal with the long-range electrostatic interactions under periodic boundary conditions^67^. The SHAKE method was used to constrain hydrogen atoms and the time step was set to 2 fs (ref. 68). The coordinates were saved every 20 ps for the subsequent analysis.

### Statistical analysis

Data were presented as means±s.e.m. The statistical differences between groups were determined by the Student’s *t*-test or one-way analysis of variance for multiple comparisons in GraphPad Pro (GraphPad, San Diego, CA). Differences were considered to be significant at *P*<0.05.

### Data availability

All other data are included within the article or Supplementary Information or available from the authors on request.

## Additional information

**How to cite this article:** Wang, Y. *et al*. Saturated palmitic acid induces myocardial inflammatory injuries through direct binding to TLR4 accessory protein MD2. *Nat. Commun.* **8,** 13997 doi: 10.1038/ncomms13997 (2017).

**Publisher's note:** Springer Nature remains neutral with regard to jurisdictional claims in published maps and institutional affiliations.

## Supplementary Material

Supplementary Information Supplementary Tables and Supplementary Figures

## Figures and Tables

**Table 1:** Biometric and echocardiographic parameters of wild-type (WT) and *Md2*^*−/−*^ (KO) mice fed control or high-fat diet for 4 months.

**Parameter**	**B6-Con (*****N*****=8)**	**B6-HFD (*****N*****=8)**	**KO-Con (*****N*****=8)**	**KO-HFD (*****N*****=8)**
Heart rate (bmp)	469.8±34.76	492.4±20.8	445.6±25.85	460.0±46.50
Fractional shortening (%)	41.92±0.75	38.13±1.34*	43.30±1.02	40.18±0.82^#^
LVEF (%)	79.38±0.79	67.98±1.21*	78.10±1.52	77.62±1.10^#^
LVID d (mm)	3.02±0.05	3.37±0.06*	3.22±0.06	3.38±0.08
LVID s (mm)	1.78±0.05	2.21±0.06*	1.85±0.08	1.88±0.05^#^
LVPW d (mm)	0.91±0.005	0.96±0.004	0.88±0.010	0.91±0.008
LVPW s (mm)	1.02±0.007	1.15±0.02*	1.04±0.01	1.05±0.01^#^
LVFW d (mm)	0.91±0.01	0.97±0.007*	0.90±0.01	0.91±0.01^#^
LVFW s (mm)	1.08±0.005	1.15±0.013*	1.03±0.014	1.07±0.016^#^
IVS d (mm)	0.92±0.013	0.95±0.006	0.88±0.008	0.90±0.013^#^
IVS s (mm)	1.07±0.003	1.16±0.016*	1.03±0.012	1.04±0.024^#^

ANOVA, analysis of variance; HFD, high-fat diet; IVS, interventricular septum; d, diastolic; KO, knockdout; LFFW, left ventricle free wall; LVEF, left ventricle ejection fraction; LVID, left ventricle internal dimension; LVPW, left ventricle posterior wall; s, systolic.

Data are reported as mean±s.e. and analysed by one-way ANOVA, *P<0.05 versus B6-LF group; ^#^*P*<0.05 versus B6-HFD group.
