# Author Correction: Saturated palmitic acid induces myocardial inflammatory injuries through direct binding to TLR4 accessory protein MD2

**DOI:** 10.1038/ncomms16185

**Published:** 2018-03-19

**Authors:** Yi Wang, Yuanyuan Qian, Qilu Fang, Peng Zhong, Weixin Li, Lintao Wang, Weitao Fu, Yali Zhang, Zheng Xu, Xiaokun Li, Guang Liang

Nature Communications 8: Article number: 13997; DOI: 10.1038/ncomms13997 (2017); Published: 01 03 2017; Updated: 03 19 2018

The original version of this Article contained an error in the Methods section entitled ‘Fatty acid-albumin complex’, where the bovine serum albumin concentration of the 5 mM palmitic acid solution used for *in vitro* experiments was incorrectly reported as being 28 μM, and should have read 627 μM. This error has now been corrected in both the PDF and HTML versions of the Article.

