# Supplementary material for: Saturated palmitic acid induces myocardial inflammatory injuries through direct binding to TLR4 accessory protein MD2
Source: Nat Commun. 2017 Jan 3;8:13997. doi: 10.1038/ncomms13997 (PMC5216130; doi:10.1038/ncomms13997)
Supplement: Supplementary Information — Supplementary Tables and Supplementary Figures [file ncomms13997-s1.pdf]

Supplementary Information File

Supplementary Table 1. List of synthesized siRNA sequences for target genes

siRNA	Species	Sequence	
Ctrl siRNA	mouse	sense	5'-UUCUCCGAACGUGUCACGUTT-3'
		Antisense	5'-ACGUGACACGUUCGGAGAATT-3'
TLR4-siRNA	mouse	sense	5'-CCUCCAUAGACUUCAAUUATT-3'
		Antisense	5'-UAAUUGAAGUCUAUGGAGGTT-3'
MD2-siRNA	mouse	sense	5'-CCCUAUUUCAAUUAGUUCUTT-3'
		Antisense	5'-AGAACUAAUUGAAAUAGGGTT-3'
MyD88-siRNA	mouse	sense	5'-CUGCGGUUCAUCACUAUAUTT-3'
		Antisense	5'-AUAUAGUGAUGAACCGCAGTT-3'
Ctrl siRNA	rat	sense	5'- UUCUCCGAACGUGUCACGUTT-3'
		Antisense	5'- ACGUGACACGUUCGGAGAATT-3'
MD2-siRNA	rat	sense	5'- CCCUAUUUCAAUUAGUUCUTT-3'
		Antisense	5'- AGAACUAAUUGAAAUAGGGTT-3'

Supplementary Table 2. Primer sequences for qPCR analysis

Gene	Species	Forward Primer	Reverse Primer
ANP	Mouse	AACCTGCTAGACCACCTGGA	TGCTTTTCAAGAGGGCAGAT
BNP	Mouse	GTCAGTCGTTTGGGCTGTAAC	AGACCCAGGCAGAGTCAGAA
TGF-β	Mouse	TGACGTCACTGGAGTTGTACGG	GGTTCATGTCATGGATGGTGC
Collagen1	Mouse	TGGCCTTGGAGGAAACTTTG	CTTGGAAACCTTGTGGACCAG
C-TGF	Mouse	ACTATGATGCGAGCCAACTGC	TGTCCGGATGCACTTTTTGC
α-MyHC	Mouse	GCCAAGACTGTCCGGAATGA	TGGAAGATCACCCGGGACTT
TNF-α	Mouse	TGATCCGCGACGTGGAA	ACCGCCTGGAGTTCTGGAA
IL-6	Mouse	GAGGATACCACTCCCAACAGACC	AAGTGCATCATCGTTGTTCATACA
IL-1β	Mouse	ACTCCTTAGTCCTCGGCCA	CCATCAGAGGCAAGGAGGAA
ICAM-1	Mouse	GCCTTGGTAGAGGTGACTGAG	GACCGGAGCTGAAAAGTTGTA
VCAM-1	Mouse	TGCCGAGCTAAATTACACATTG	CCTTGTGGAGGGATGTACAGA
MyD88	Mouse	AGGACAAACGCCGGAACTTTT	GCCGATAGTCTGTCTGTTCTAGT
MD2	Mouse	GACGCTGCTTTCTCCCATA	CATTGGTTCCCCTCAGTCTT
TLR4	Mouse	TGTTCTTCTCCTGCCTGACA	TGTCATCAGGGACTTTGCTG
β-actin	Mouse	TGCACCACCAACTGCTTAG	GGATGCAGGGATGATGTTC
ICAM-1	Rat	AGATCATACGGGTTTGGGCTTC	TATGACTCGTGAAAGAAATCAGCTC
VCAM-1	Rat	TTTGCAAGAAAAGCCAACATGAAAG	TCTCCAACAGTTCAGACGTTAGC
TGF-β	Rat	GGACTACTA CGCCAA AGA AG	TCA AAAGACAGCCACTCAGG
Collagen1	Rat	GAGCGGAGAG TACTGGATCGA	CTGACCTGTCTCCAT- GTTGCA
ANP	Rat	CTGCTAGACCACCTGGAGGA	AAGCTGTTGCAGCCTAGTCC
BNP	Rat	GATCCAGGAGAGACTTCGAAA	CGGTCTATCTTCTGCCCAA
IL-1β	Rat	CACCTCTCAAGCAGAGCACAG	GGGTTCCATGGTGAAGTCAAC
TNF-α	Rat	TACTCCCAGGTTCTCTTCAAGG	GGAGGCTGACTTTCTCCTGGTA
IL-6	Rat	GAGTTGTGCAATGGCAATTC	ACTCCAGAAGACCAGAGCAG
Cox2	Rat	CGGAGGAGAAGTGGGGTTTAGGAT	TGGGAGGCACTTGCGTTGATGG
CTGF	Rat	GCCTGTTCCAAGACCTGT	GGATGCACTTTTTGCCCTTCTTA
MHC	Rat	GAGGAGAGGGCGGACATT	ACTCTTCATTCAGGCCCTTG
β-actin	Rat	ATCGTGGGCCGCCCTAGGCACC	CTCTTTAATGTCACGCACGATTTC

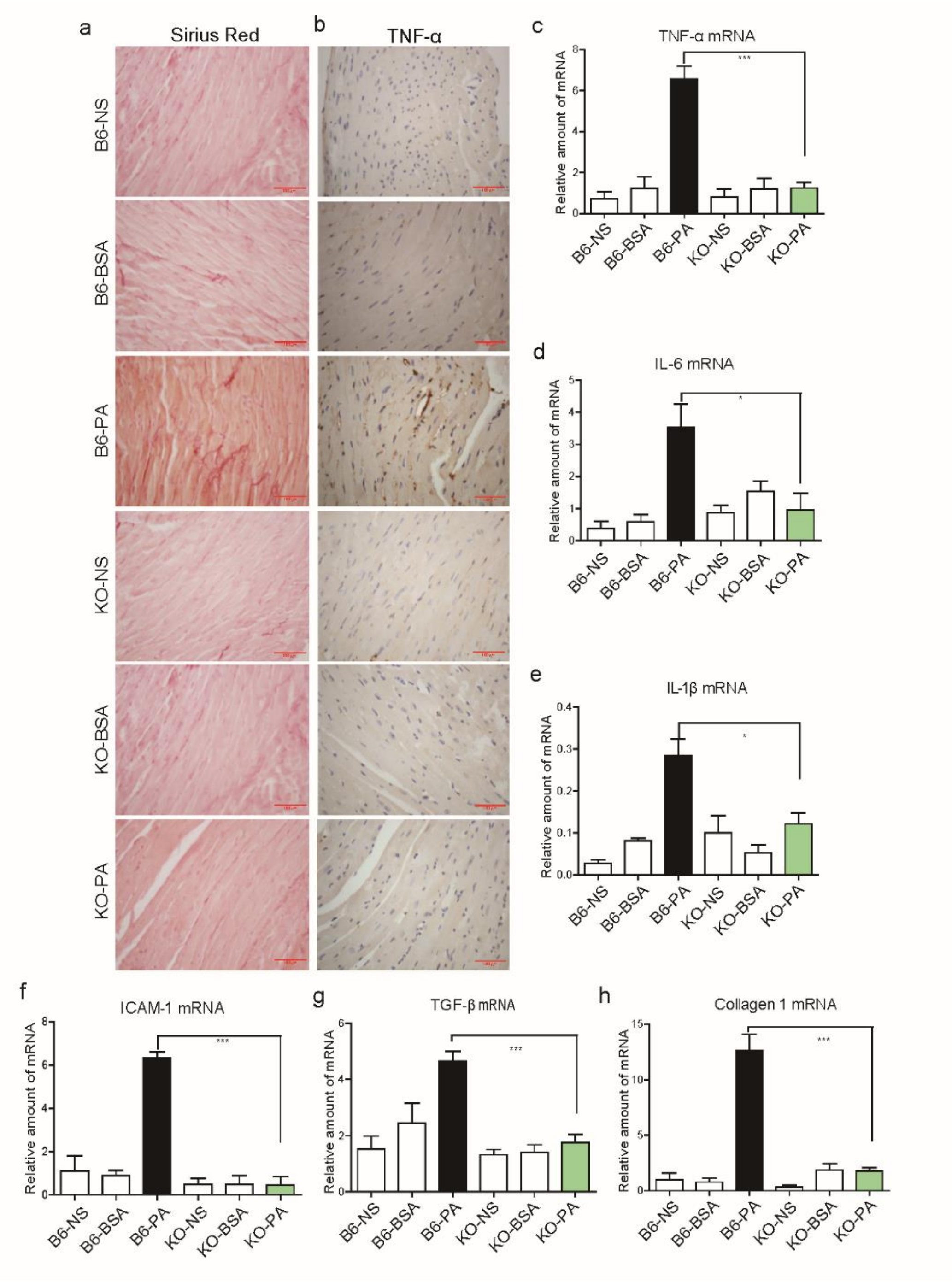

Supplementary Figure 1. ***Md2* knockout protects against myocardial inflammatory injury in response to PA challenge**

Wild-type (B6) or *Md2-/-* mice (KO) were challenged with 5 mM PA, normal saline (NS) or 5% BSA (vehicle control) twice daily for 7 days by intravenous injection (n = 8). (**a**) Representative micrograph of Sirius Red staining for fibrosis in heart tissues; scale bars = 100 μm. (**b**) Representative immunohistochemical detection of TNF-α in heart tissues; scale bars = 100 μm. (**c**-**f**) Total RNA isolated from heart tissue was used for analysis of pro-inflammatory genes. Graphs show relative mRNA values of TNF-α (c), IL-6 (d), IL-1β (e), and ICAM-1 (f). (**g** and **h**) mRNA analysis of genes associated with tissue remodeling. mRNA data were normalized to housekeeping gene β-actin; the relative values reported as mean ± s.e.m. and analyzed by Student's *t*-test; * $p < 0.05$, *** $p < 0.001$, compared with B6-PA group.

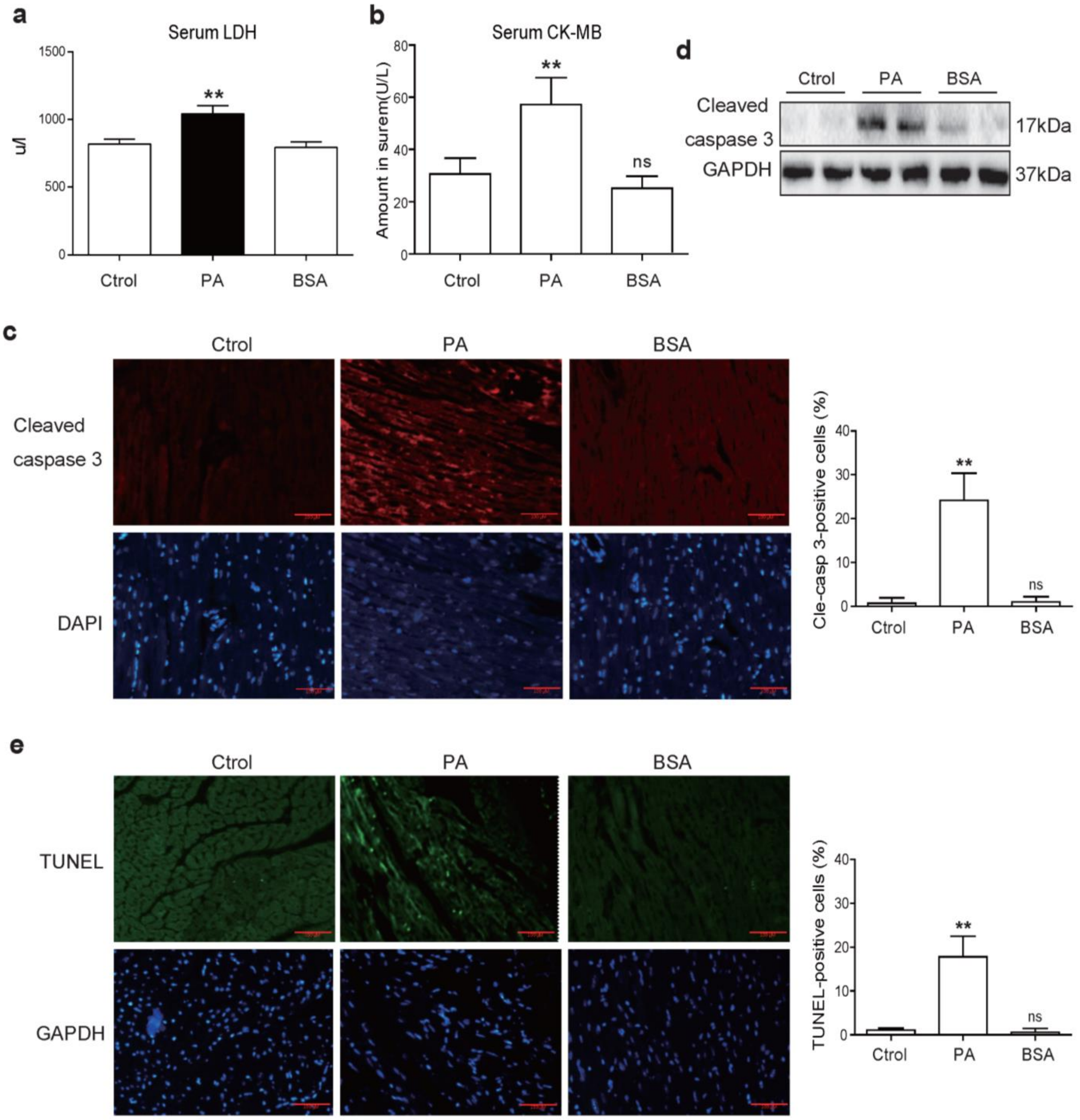

Supplementary Figure 2. PA challenge induces cell apoptosis in the heart

Wild-type (B6) mice were challenged with 5 mM PA, normal saline (Ctrol) or 5% BSA (vehicle control) twice daily for 7 days by intravenous injection (n = 8). (**a** and **b**) Serum lactate dehydrogenase (LDH) and CK-MB levels. (**c**) Cleaved-caspase 3 levels in heart tissues of mice challenged with PA. Representative immunohistochemical analysis (cleaved caspase 3 = red, nuclei = blue), scale bars = 100 µm; and quantification of cleaved-caspase 3 positive cells. (**d**) Western blot analysis of cleaved caspase 3 in heart tissues (GAPDH used as loading control; representative of 3 separate determinations). (**e**) Cardiac cell death as assessed by TUNEL staining (green). Tissues were counterstained with DAPI, scale bars = 100 µm. Quantification of TUNEL positive cells is provided on the right. Data in (a-b) and (c-d) are reported as mean ± s.e.m. and analyzed by One-way ANOVA, ** $p < 0.01$,PA group was compared to control (ctrol or BSA); ns = not significant compared to ctrol.

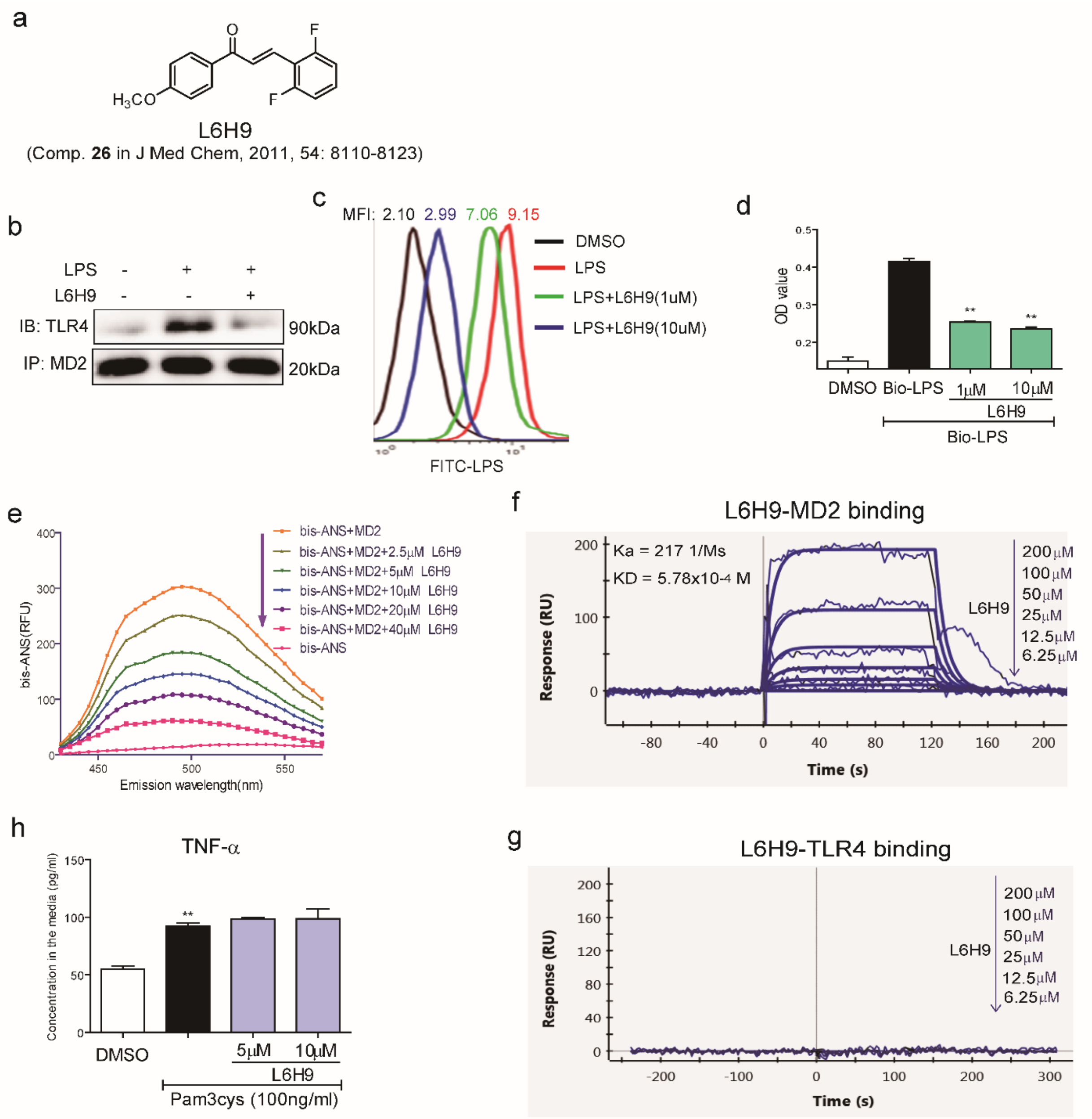

Supplementary Figure 3. L6H9 interactions with MD2

(**a**) Chemical structure of L6H9. (**b**) Representative Western blot of co-immunoprecipitated TLR4/MD2 complex with or without 10 µM L6H9 pretreatment of macrophages stimulated with 0.5 µg mL^{-1} LPS (n = 3). (**c**) Representative flow cytometric analysis showing cell surface binding of FITC-conjugated LPS (LPS-FITC) in the presence or absence of L6H9 (MFI = mean fluorescence intensity; n = 3). (**d**) Effects of L6H9 on binding of biotinylated-LPS (bio-LPS) to human recombinant MD2 (rhMD2) preabsorbed onto ELISA plates, OD values reported as mean ± s.e.m. and analyzed by Student's *t*-test; ** p < 0.01, compared to bio-LPS group; n = 3. (**e**) Representative fluorescence graph showing effects of L6H9 on the direct binding of bis-ANS, a fluorescence probe, with rhMD2 (RFU = relative fluorescence units; n = 3). (f) Representative graph from surface plasmon resonance spectroscopy (SPR) analysis of L6H9 binding to rhMD2 (Ka and KD values are indicated on the upper left; n = 3). (g) Representative SPR analysis showed no direct interaction between L6H9 and rhTLR4 (n = 3). (**h**) Effects of L6H9 on TNF-α secretion by macrophages stimulated with the TLR2 agonist, Pam3cys; data reported as mean ± s.e.m. and analyzed Student's *t*-test, ** p < 0.01 compared with DMSO control, n = 3).

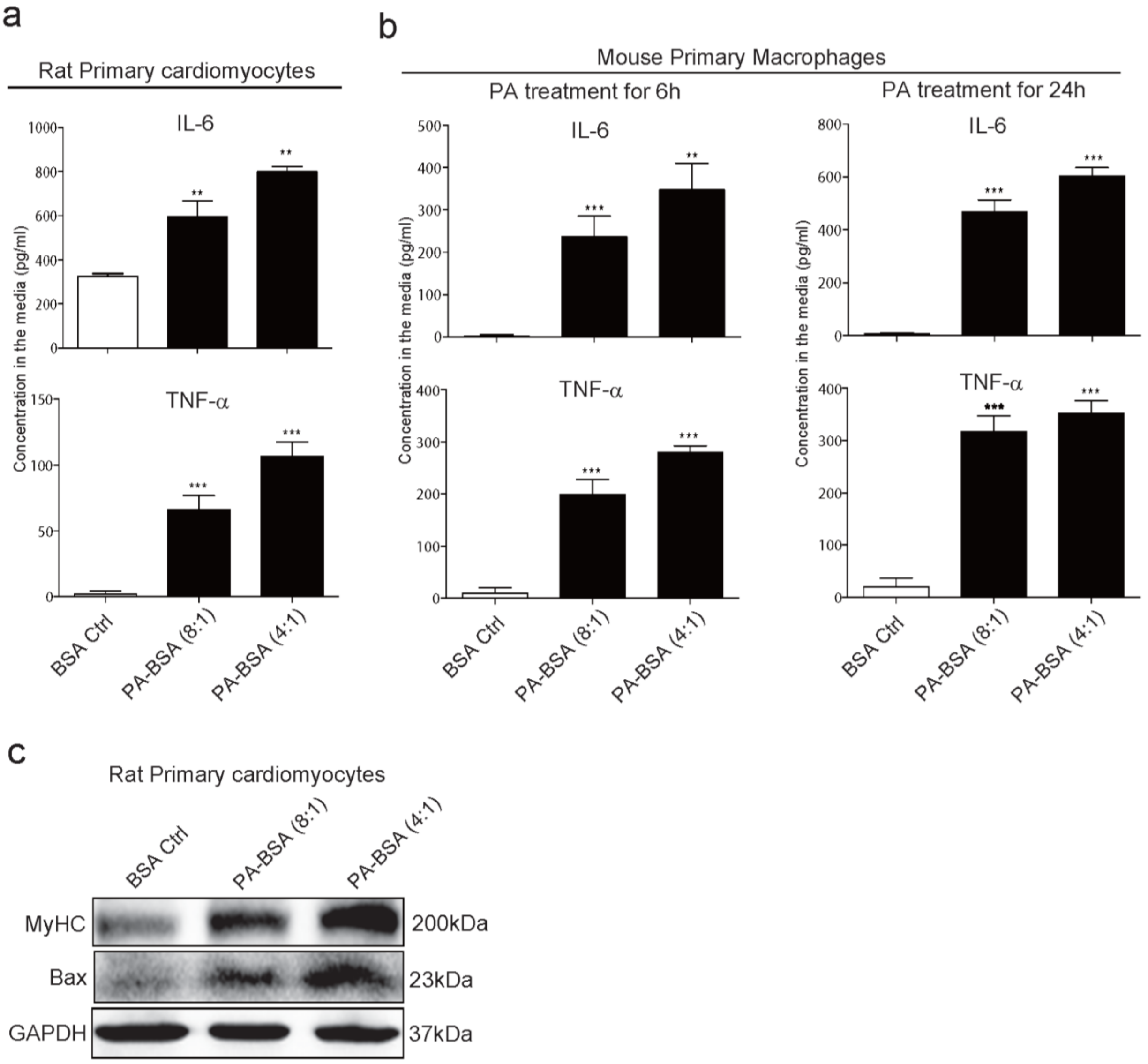

Supplementary Figure 4. Comparison of PA:BSA ratios of 4:1 and 8:1 cardiomyocyte cytokine production and injury

(**a**) Rat primary cardiomyocytes were stimulated with 500 µM PA in BSA solution with a PA:BSA ratio of 4:1 or 8:1 for 24 h; (**b** and **c**) Mouse primary macrophages were stimulated with 100 µM PA in BSA solution with a PA:BSA ratio of 4:1 or 8:1 for 6 h or 24 h, respectively. Conditioned media were collected from the two cell types, and TNF-α and IL-6 were measured by ELISA. Data are reported as mean ± s.e.m. pg mL^{-1} and analyzed by Student's *t*-test, **p<0.01, ***p<0.001 compared to BSA control; n = 3. (**d**) Rat primary cardiomyocytes were stimulated with 500 µM PA in BSA solution with a PA:BSA ratio of 4:1 or 8:1 for 24 h. Protein levels of MyHC and Bax in cell lysates were measured by Western Blot analysis.

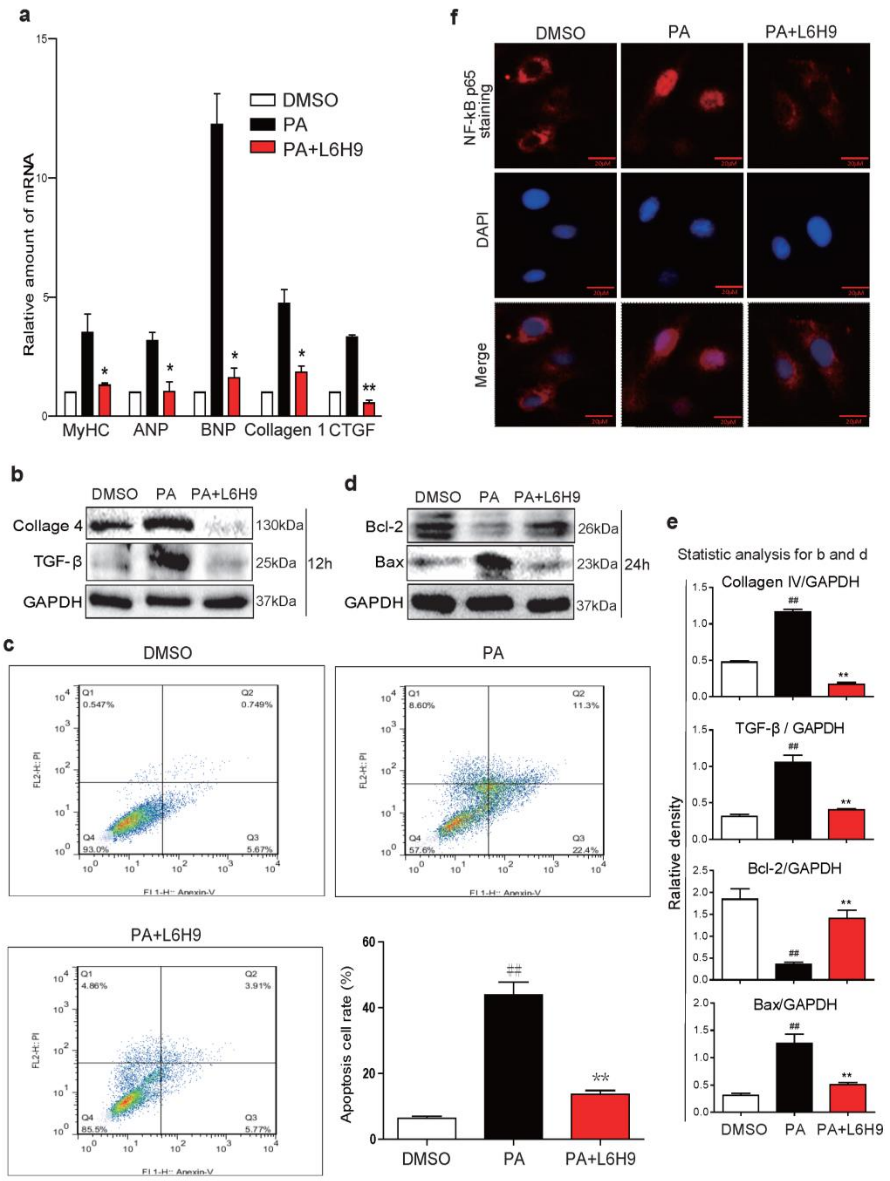

Supplementary Figure 5. MD2 inhibition by L6H9 prevents hypertrophy and apoptosis in cultured cardiomyocytes

(**a**) H9C2 cells were pretreated with L6H9 (10 μM) before stimulation with 500 μM PA for 24 h. Real-time qPCR determination of hypertrophic markers (myosin heavy chain, MyHC; atrial natriuretic peptide, ANP; and brain natriuretic peptide, BNP) and fibrosis markers (collagen 1 and connective tissue growth factor, CTGF). mRNA values were normalized housekeeping gene β-actin and reported as normalized to DMSO control group, n = 3 (. (**b**) Representative Western blot of collagen IV and TGF-β (n=3). (**c**) Effects of L6H9 (10 μM) pretreatment on apoptosis in H9C2 cells induced by 500 μM PA for 24 h. Representative flow cytometric analysis of FITC-Annexin V and propidium iodide (PI); quantification of flow cytometric data for apoptotic cells, n = 3. (**d**) Representative Western blot of apoptosis-related proteins Bcl2 and Bax in H9C2 cells exposed to PA with or without L6H9 pretreatment; n = 3. (**e**) Densitometric quantification of Western blot data shown in panels b and d. Data in (a, c, e) are reported as mean ± s.e.m. and analyzed by Student's *t*-test,$^{##}$ $p < 0.01$, compared to DMSO group; * $p < 0.05$, ** $p < 0.01$ compared to PA group. (**f**) Representative immunofluorescent localization of NF-κB p65 subunit in H9C2 cells, scale bars = 20 μm, (p65 = red; nuclei = blue; n = 3 separate determinations).

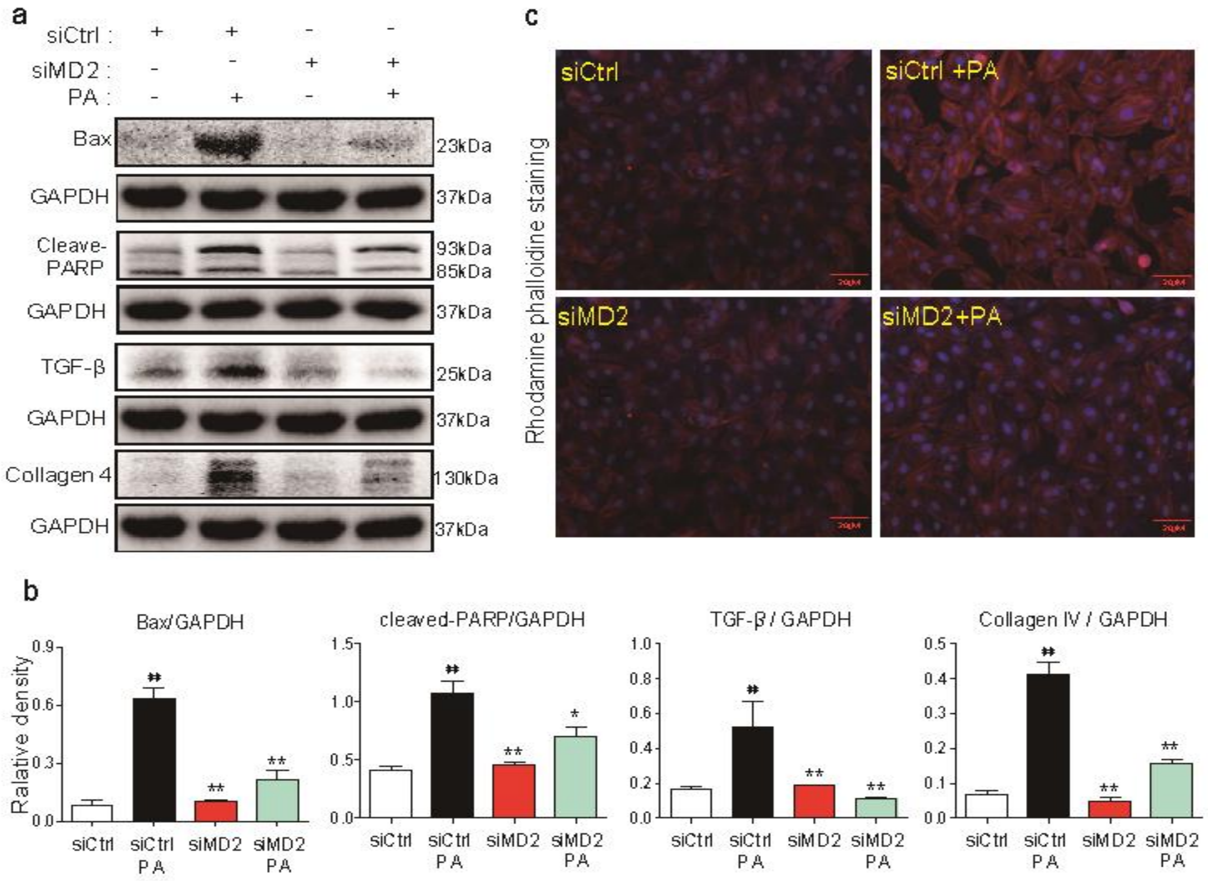

Supplementary Figure 6. ***Md2*** **knockdown prevents cardiomyocyte apoptosis and fibrogenic factor expression**

(**a**) Representative Western blot analysis of apoptosis-related proteins (Bax and cleaved-poly ADP ribose polymerase (PARP)) and pro-fibrosis proteins (collagen 4 and TGF-β) in cells stimulated with 500 µM PA for 24 h. (**b**) Densitometric quantification of Western blot data shown in panel a; values reported as mean ± s.e.m. and analyzed by Student's *t*-test, $^{\#\#}$ $p < 0.01$, compared to siCtrl group; * $p<0.05$, ** $p < 0.01$ compared to siCtrl-PA group, n = xx. (**c**) Representative fluorescence staining of cells stimulated with PA for 12 h showing F-actin changes detected with rhodamine-phalloidin (red). Cells were counterstained with DAPI (blue), n = 3, scale bars = 20 µm.

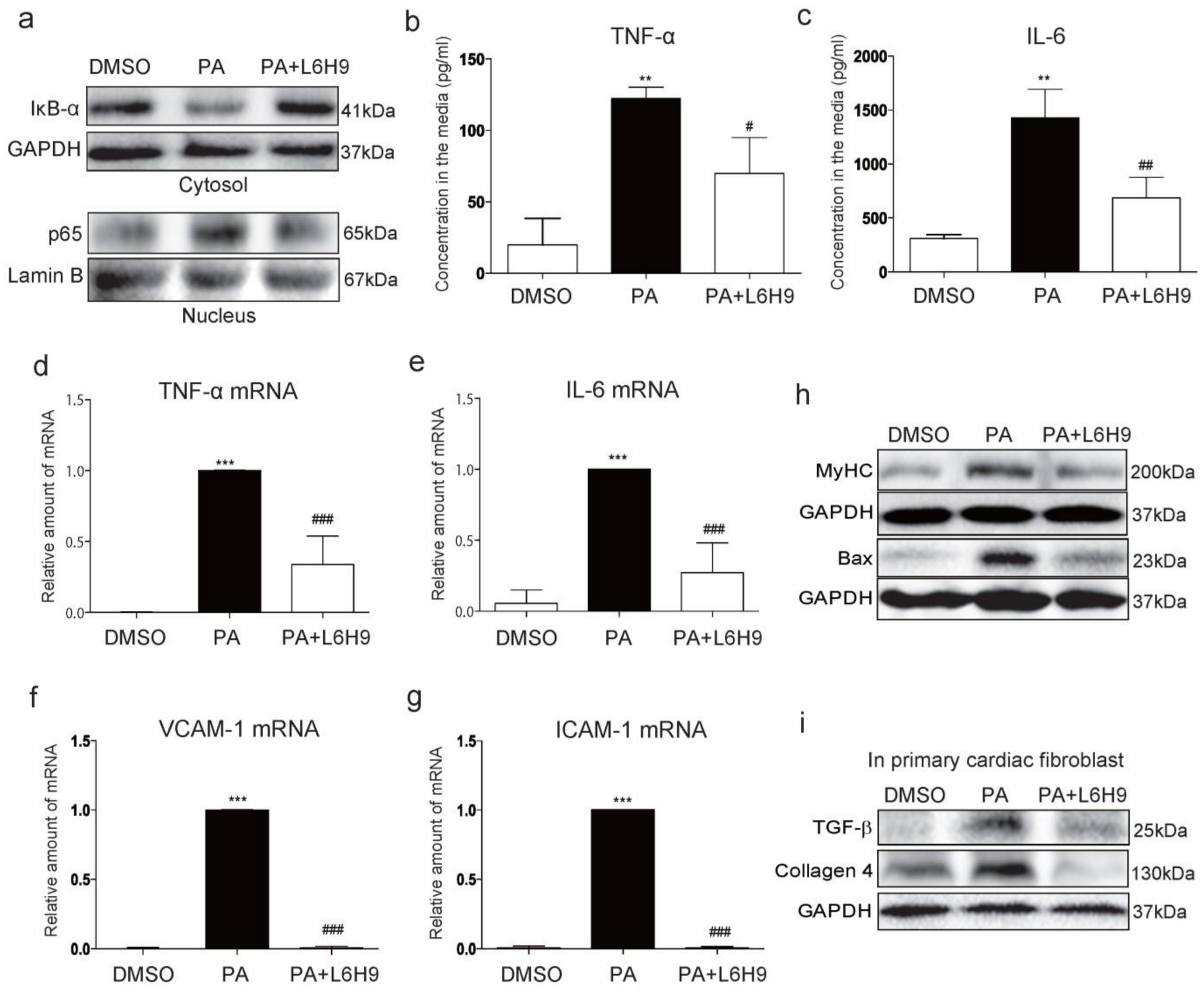

Supplementary Figure 7. Effect of MD2 inhibitor, L6H9, on primary rat cardiomyocyte inflammatory injuries

Primary neonatal rat cardiomyocytes were treated identically to H9C2 (Supplementary Figure 5) and assayed for pro-inflammatory cytokines and fibrogenic factors. (**a**) Western blot detection of inhibitor of κB (IκB) in the cytosol and p65 subunit of NF-κB in nuclear fraction of cells exposed to 500 µM PA (n = 3, GAPDH and lamin B as respective loading controls). (**b** and **c**) Cytokine protein levels were measured in condition media of primary rat cardiomyocytes treated with 500 µM PA with or without 10 µM L6H9 pre-treatment; values normalized to media volume as pg mL^{-1}, n = 3. (**d-g**) qPCR analysis of mRNA levels of pro-inflammatory genes and adhesion molecules; mRNA values normalized to housekeeping gene β-actin and relative levels are reported for TNF-α (d), IL-6 (e), VCAM-1 (f), and ICAM-1 (g), n = 3. (**h)** Representative Western blot analysis of hypertrophic factor MyHC and apoptosis-related protein Bax in primary cardiomyocytes (n = 3, GAPDH used as loading control).. (**i**) Representative Western blot analysis of collagen 4 and TGF-β in primary cardiac fibroblasts stimulated with PA with or without MD2 inhibitor L6H9 pre-treatment; n=3. Data in (b-g) are reported as mean ± s.e.m. and analyzed by Student's *t*-test, ** $p < 0.01$, *** $p < 0.001$ compared to DMSO group; $^{\#}$ $p < 0.05$, $^{\#\#}$ $p < 0.01$, $^{\#\#\#}$ $p < 0.001$ compared to PA group.

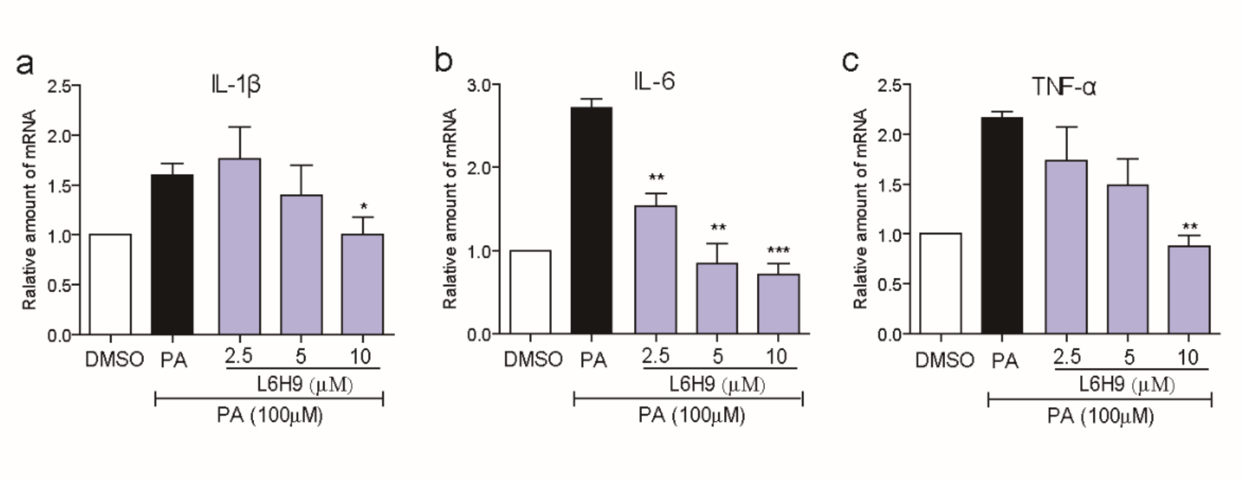

Supplementary Figure 8. L6H9 inhibits PA-induced inflammatory cytokine expression in macrophages

Mouse primary macrophages were treated with 100 µM PA for 6 h with or without 1 h pretreatment with L6H9. Relative mRNA values are reported as mean ± s.e.m. and analyzed by Student's *t*-test for IL-1β (**a**), IL-6 (**b**), and TNF-α (**c**) (* $p < 0.05$, ** $p < 0.01$, *** $p < 0.001$ compared to PA group; n = 3).

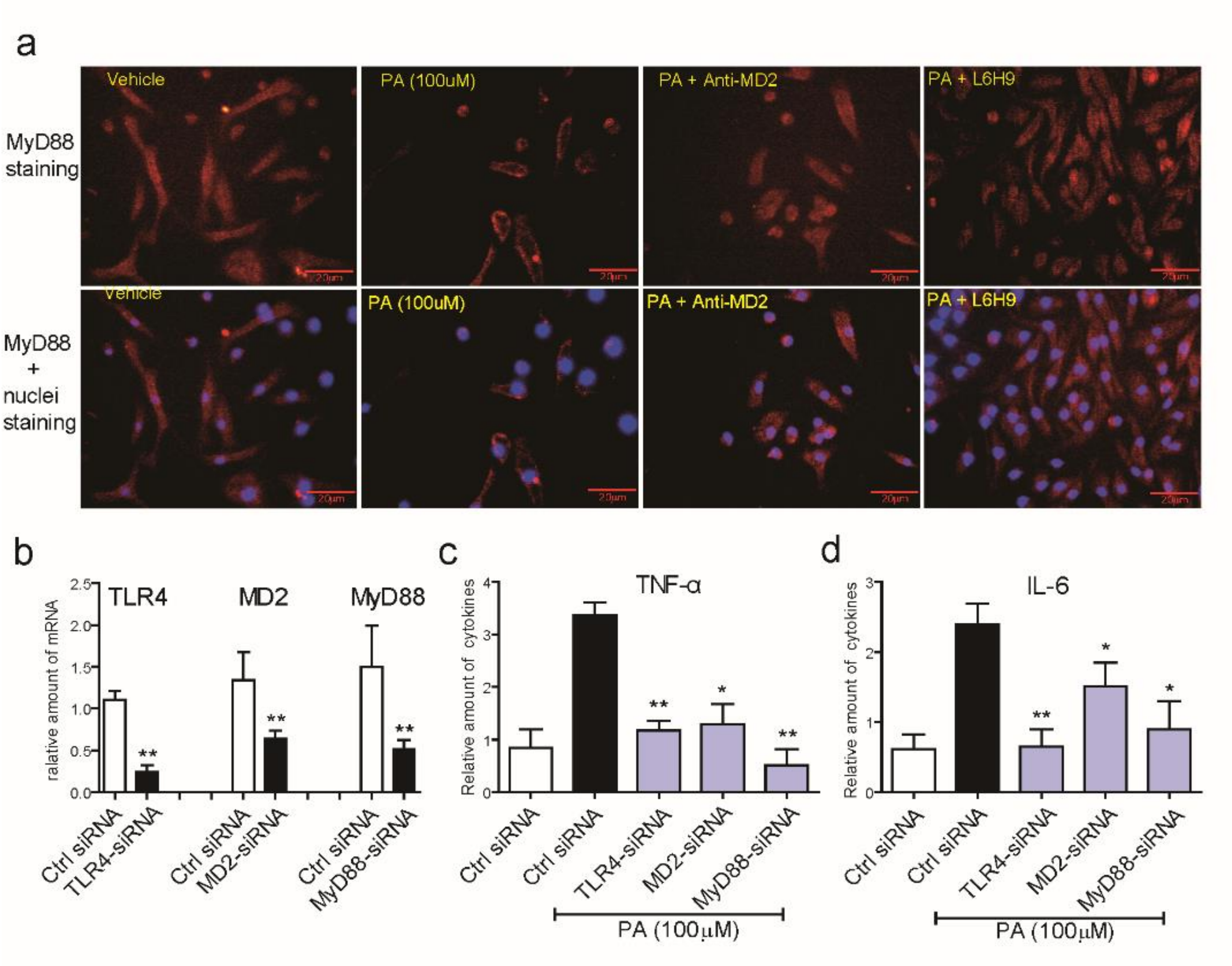

Supplementary Figure 9. MD2 is required for the formation of MD2/TLR4-MyD88 complex and cytokine expression

(**a**) Immunofluorescence staining of macrophages for MyD88. Cells were stimulated with PA (100 µM for 1 h) after 1 h pretreatment with the MD2 neutralizing antibody (anti-MD2) (100 ng mL^{-1}) or with L6H9 (10 µM) (scale bars = 20 µm; n = 3). (**b**) mRNA levels of MD2-TLR4 pathway following knockdown of each member of the complex. Transfections were carried out with siRNA against MD2, TLR4, or MyD88 (** $p < 0.01$ compared to control siRNA; n = 3). (**c** and **d**) Effect of knocking down MD2,TLR4, or MyD88 on PA-stimulated (100 µM for 6h). Relative mRNA values shown for TNF-α (**c**) and IL-6 (**d**). Data for (b-d) are reported as mean ± s.e.m. and analyzed by Student's *t*-test, * $p < 0.05$, ** $p < 0.01$ when compared to ctrl-siRNA group (b), or compared with PA+ctrl-siRNA group (c-d); n = 3).

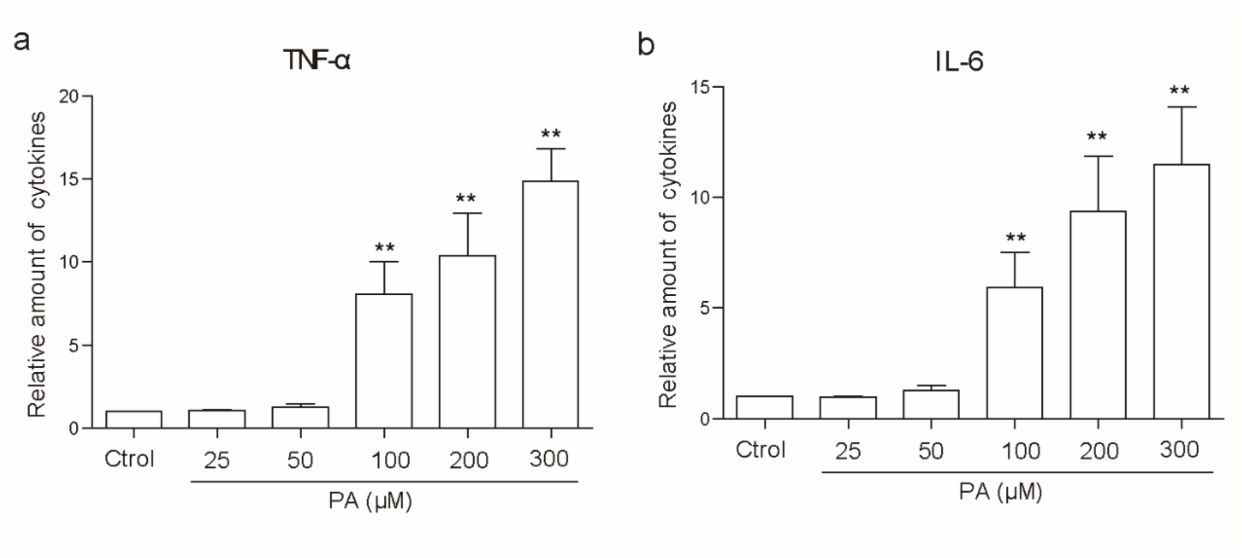

Supplementary Figure 10. PA induces macrophage cytokine expression in a dose-dependent manner

Primary macrophages were treated with different concentrations of PA for 24 h. Protein levels of a) TNF-α and b) IL-6 in culture media were measured by ELISA kits to determine the minimum concentration of PA needed to elicit cytokine induction; values reported as mean ± s.e.m. and analyzed by Student's *t*-test, ** $p < 0.01$ compared to Ctrol group, n = 3.

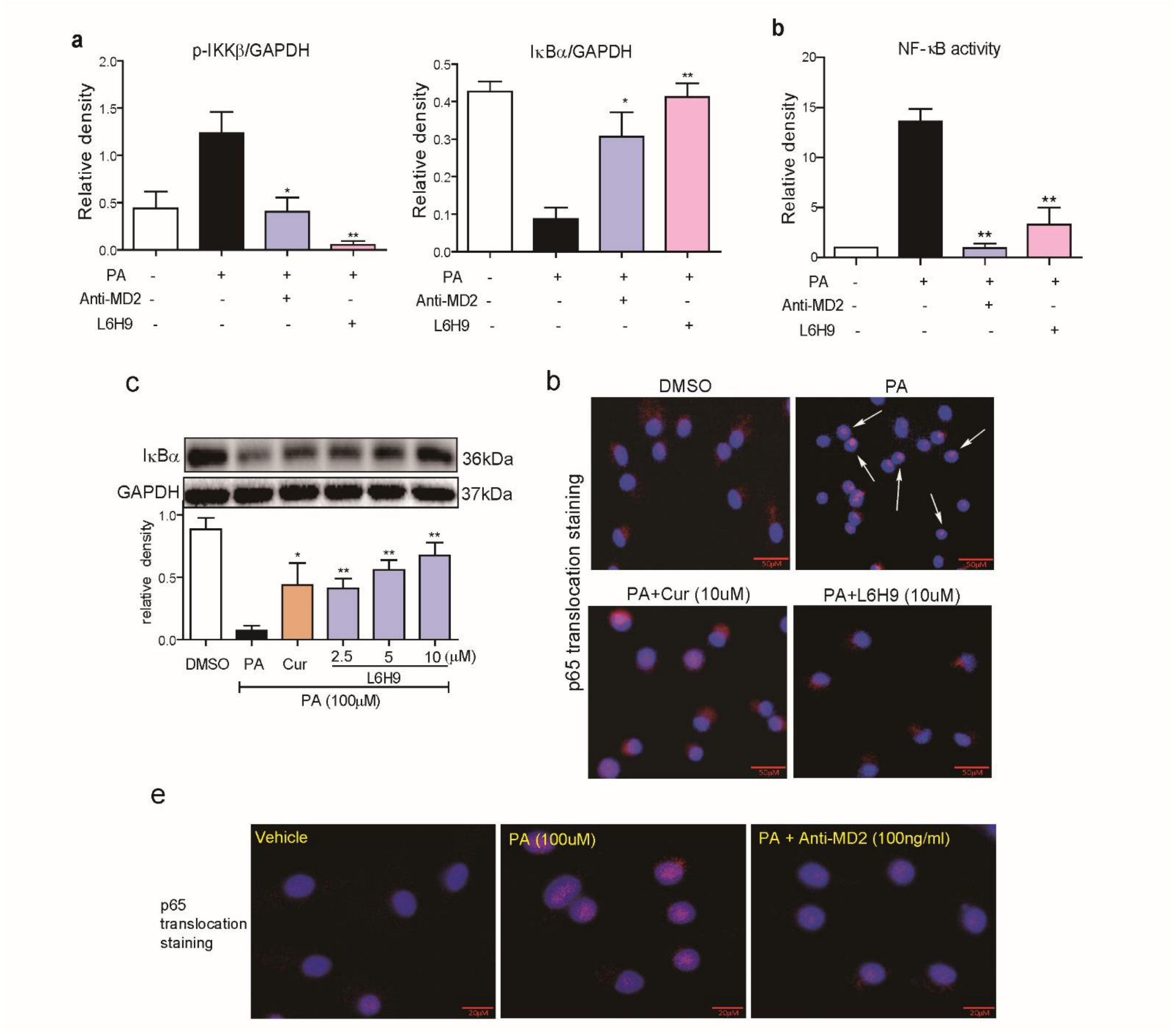

Supplementary Figure 11. MD2 blockade prevents NF-κB activation in macrophages

(**a** and **b**) Densitometric quantification of for p-IKKβ and IκB-α presented in Figure 3g, and for NF-κB DNA-binding activity in Figure 3h, n = 3. (**c-e**) MD2 was blocked by L6H9 at indicated concentrations or MD2 neutralizing antibody anti-MD2 (100 ng mL^{-1}). Curcumin (Cur) at 10 μM was used as comparison. Cells were then stimulated with PA (100 μM) for time periods as indicated and evaluated. (**c**) Following 30 min of PA stimulation, macrophages were prepared for Western blot analysis of IκB-α; shown is a representative blot; densitometric quantification of bands shown below, n = 3; . Data in (a-c) reported as mean ± s.e.m. and analyzed by Student's *t*-test, * $p < 0.05$, ** $p < 0.01$ compared to PA group). (**d** and **e**) NF-κB nuclear translocation was evaluated by immunofluorescence localization of NF-κB p65 subunit (red) after 1 h of PA stimulation of macrophages (nuclei = blue; representative of 3 separate experiments; scale bars = 50 μm). (**d**) p65 localization in cells pretreated with L6H9 or Cur. (**e**) p65 localization in cells treated with anti-MD2, scale bars = 20 μm.

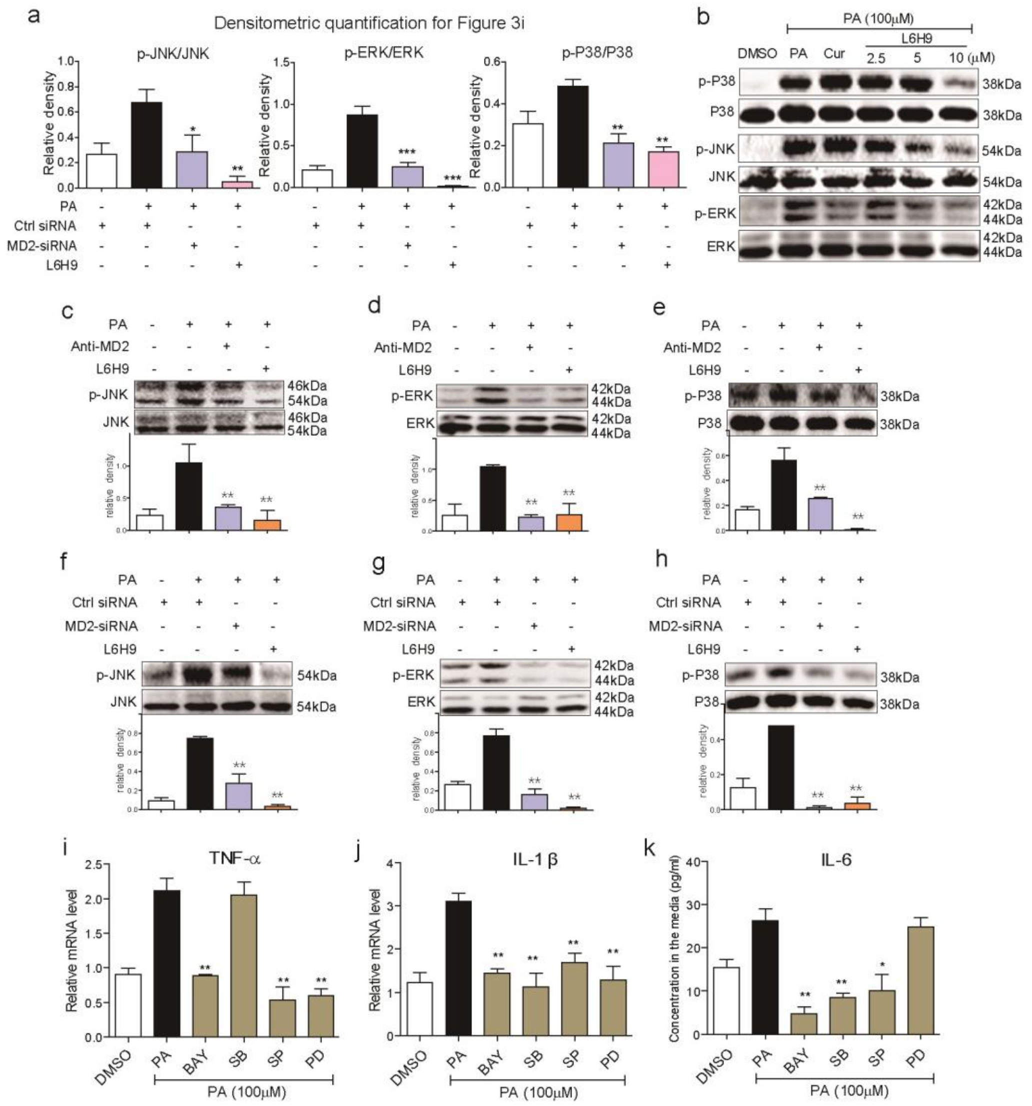

Supplementary Figure 12. MD2 blockade prevents PA-induced activation of MAPK signaling **(a)** Densitometric quantification of phosphorylated JNK, ERK, and P38 shown in Figure 3i; values normalized to total unphosphorylated protein. (**b**-**e**) MD2 blockade was made with L6H9 (2.5-10 μM) (b), MD2 neutralizing antibody (anti-MD2) (c-e), or siRNA transfections (*Md2*-siRNA) (f-h) in mouse primary macrophages. Cells were then stimulated with PA (100 μM for 30 min), and phosphorylation status of MAPKs determined. (**b**) Representative Western blot analysis showing effects of increasing concentrations of L6H9 on PA-induced phosphorylation of p38, JNK and ERK. (**c**-**e**) Effects of anti-MD2 or L6H9 (10 μM) on PA-stimulated phosphorylation of JNK (b), ERK (c), and P38 (d). (**f**-**h**) Effects of MD2-siRNA or L6H9 (10 μM) on PA-stimulated phosphorylation of JNK (e), ERK (f), and P38 (g) (ctrl = control siRNA; shown are relative band density values normalized to unphosphorylated corresponding protein). (**i**-**k**) Effects of 1 h pre-treatment with pharmacological inhibitors of MAPK signaling proteins and NF-κB on PA-stimulated (100 μM for 6 h) mRNA upregulation. Shown mRNA values are normalized to housekeeping gene β-actin l for TNF-α (**i**) and IL-1β (**j**), and secreted IL-6 (**k**) in mouse primary macrophages. [Inhibitors: BAY = 100 μM BAY 11-7082, NF-κB inhibitor; SB = 50 μM SB203580, P38 inhibitor; PD = 75 μM PD98059, ERK inhibitor; SP = 10 μM SP600125, JNK inhibitor] Data in (a) and (c-k) are reported as mean ± s.e.m. and analyzed by Student's *t*-test, * $p < 0.05$, ** $p < 0.01$, *** $p < 0.001$ when compared to PA alone or PA+siCtrl group; n = 3.

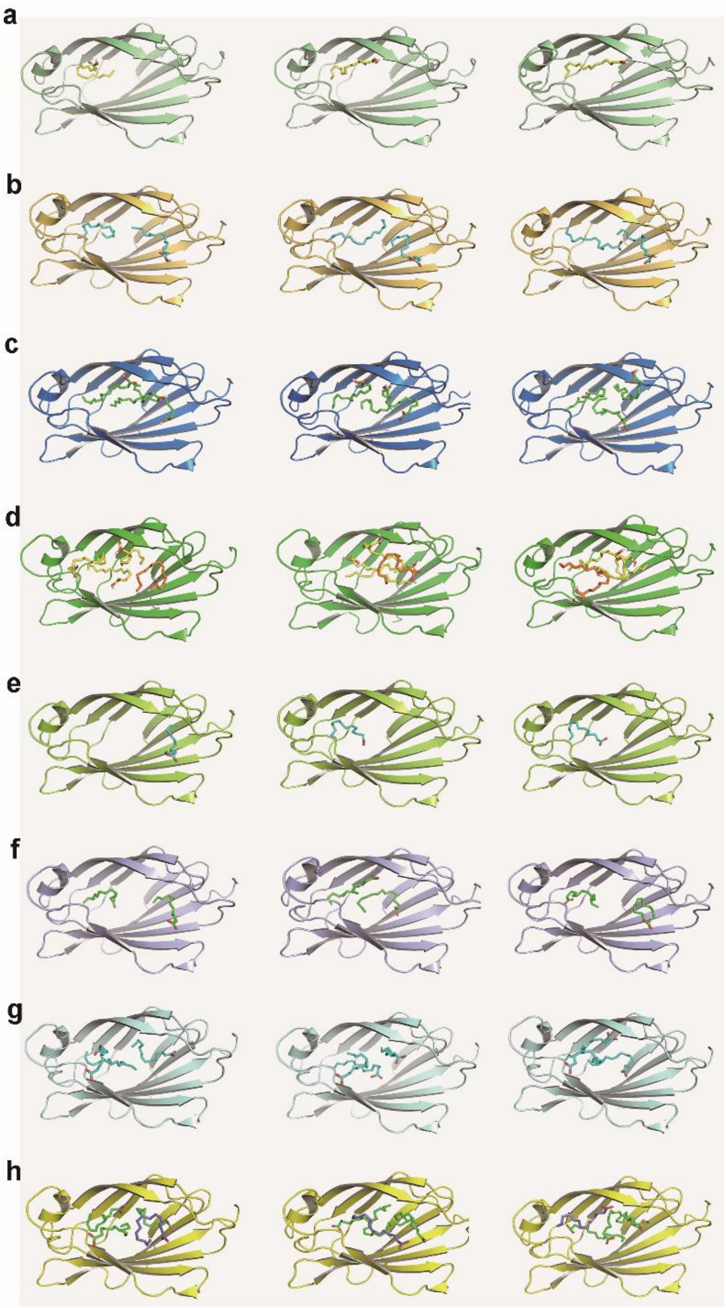

Supplementary Figure 13. Molecular docking simulation of MA or PA on MD2 based on multiple ligand simultaneous docking (MLSD) analysis

Molecular docking analysis of MA or PA within cavity of MD2 using the multiple ligand simultaneous docking program to predict binding poses at MD2:fatty acid binding ratios of 1:1, 1:2, 1:3, and 1:4 (see Methods). Shown is a representative 1 of 3 binding poses of MA or PA binding within MD2. (**a**): MD2/PA, 1:1; (**b**): MD2/PA, 1:2; (**c**): MD2/PA, 1:3; (**d**): MD2/PA, 1:4; (**e**): MD2/MA, 1:1; (**f**): MD2/MA, 1:2; (**g**): MD2/MA, 1:3; (**h**): MD2/MA, 1:4. Each binding pose includes 3 representative conformations. Up to 3 molecules of PA (or MA) bound within the binding pocket of MD2, with appropriate positioning of hydrophobic carbon tail of the fatty acids oriented towards inside the MD2 hydrophobic pocket, with the hydrophilic carboxyl heads (red dot) towards outside the pocket. With 4 molecules of PA (or MA), one of the PA (orange) and MA (purple) presented with unnatural binding poses, appearing to be located outside the MD2 binding pocket.

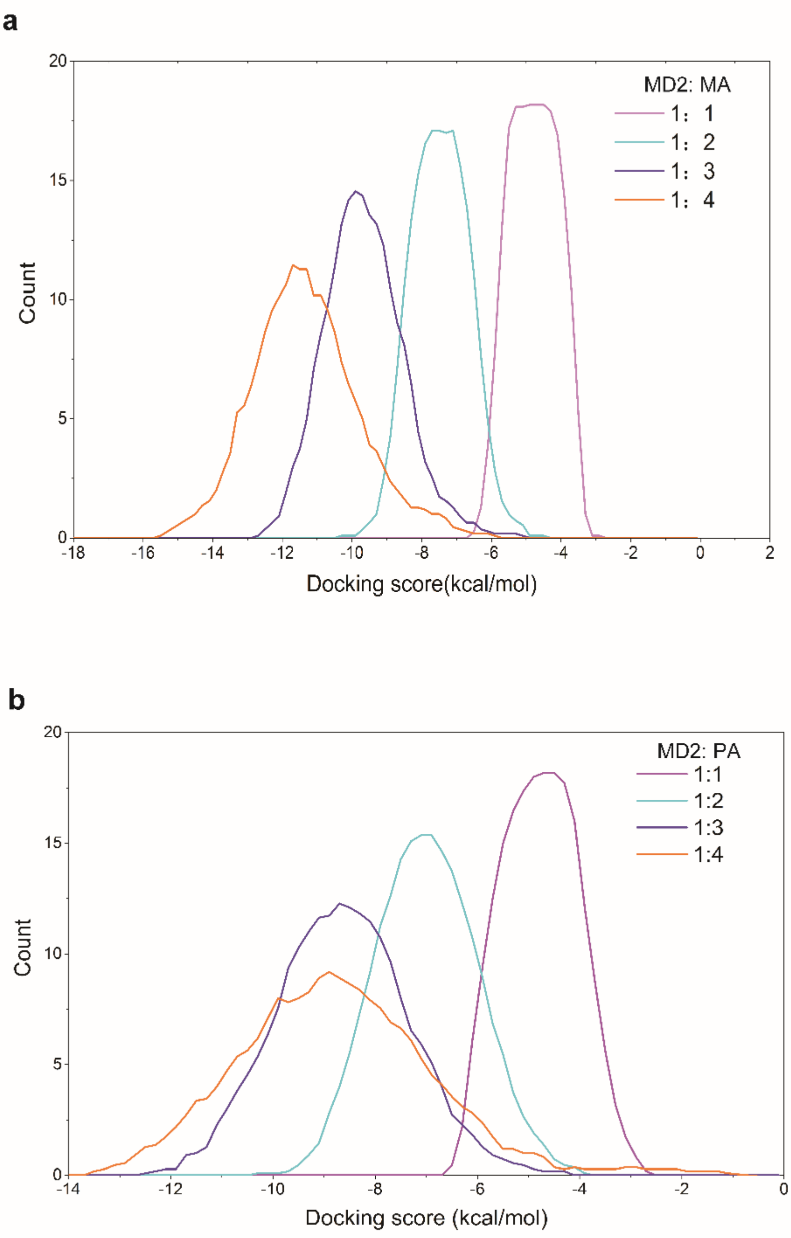

Supplementary Figure 14. Distribution of predicted free energy of binding

The multiple ligand simultaneous docking (MLSD) program was used to predict the docking scores (free energy of binding) of binding poses of MA or PA in the active site of MD2 (see Methods). Shown are the distribution curves of 200 docking scores by (**a**) MA and (**b**) PA at MD2:fatty acid binding ratios of 1:1 (violet), 1:2 (cyan), 1:3 (purple), and 1:4 (orange). An increasing binding ratio corresponded with decreasing docking scores, with the lowest score at 1:3 and 1:4 binding ratios. However, it should be noted that the 1:4 ratio for either PA or MA yielded unnatural binding poses with MD2 (see Supplementary Fig. 13).

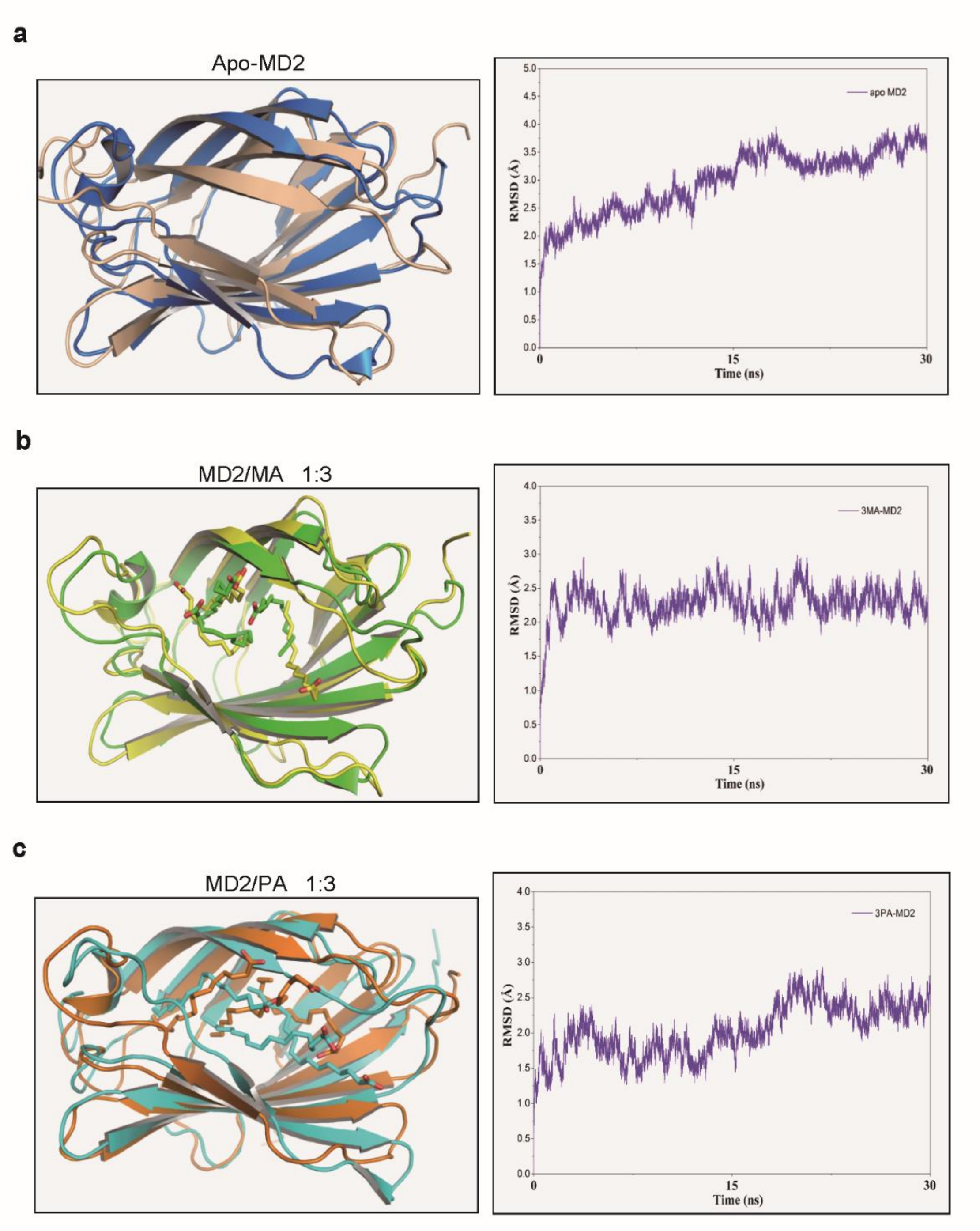

Supplementary Figure 15. Molecular dynamic simulation of binding poses

(**a**) Crystal structure of apo-MD2 (with ligand 2e56 removed); (left panel) initial snapshot of crystal structure (blue) superimposed with structure at 30 ns (wheat); (right panel) time evolution of the RMSD (root mean square deviation) of apo-MD2. (**b**) Binding pose of 3 molecules of MA with MD2; (left panel) initial docked structure (from the crystal structure of PDB ID 2E56, green) superimposed with structure at 30 ns (yellow); (right panel) time evolution of the RMSD of MA-MD2. (**c**) Binding pose of 3 molecules of PA with MD2; (left panel) initial docked structure (orange) superimposed with structure at 30 ns (cyan); (right panel) time evolution of the RMSD of MA-MD2. Red dot indicates the hydrophilic head of fatty acid. Analysis indicated that PA-MD2 and MA-MD2 structures showed similar structural stability within 30 ns, with RMSD values of ~2.5Å.

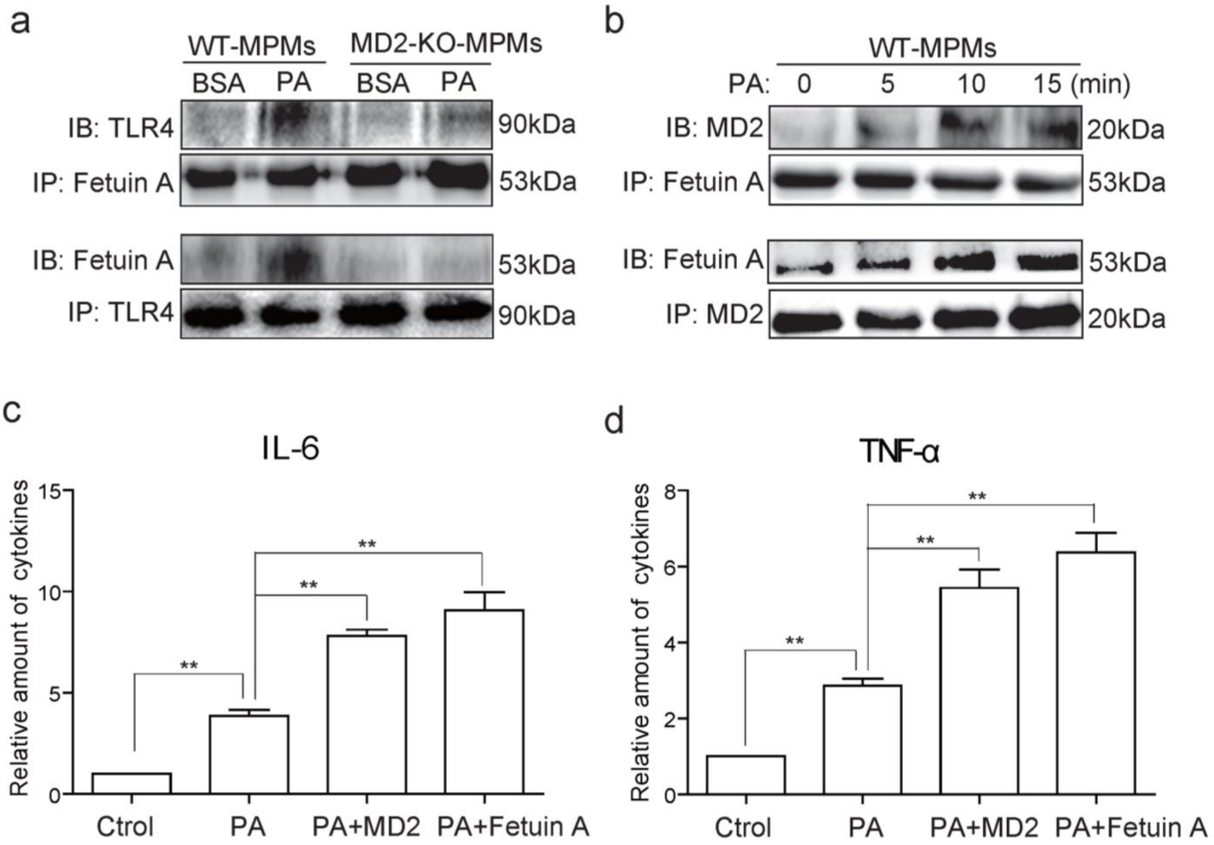

Supplementary Figure 16. Fetuin A cooperates in PA-induced TLR4 activation and cytokine production but requires MD2.

Effects of exogenous Fetuin A on MD2-TLR4 signaling in primary macrophages (**a**) MPMs were stimulated by 100 µM PA for 15 min. Fetuin A-TLR4 association was determined by co-immunoprecipitation (IP) and Western blot (IB) analysis of macrophages isolated from wild-type (B6) and *Md2*$^{-/-}$ (KO) mice (representative of 3 independent determinations). (**b**) Effect of 100 µM PA on MD2-Fetuin A association in primary macrophages isolated from wild-type mice. Lysates were analyzed following PA stimulation for different time periods. (**c** and **d**) The protein levels of IL-6 and TNF-α were measured in primary macrophages cultured in serum-free media following exposure to PA with or without exogenous Fetuin A or MD2 protein (n = 3; values reported as mean ± s.e.m. and analyzed by Student's *t*-test; ** $p < 0.01$ compared to the PA group).

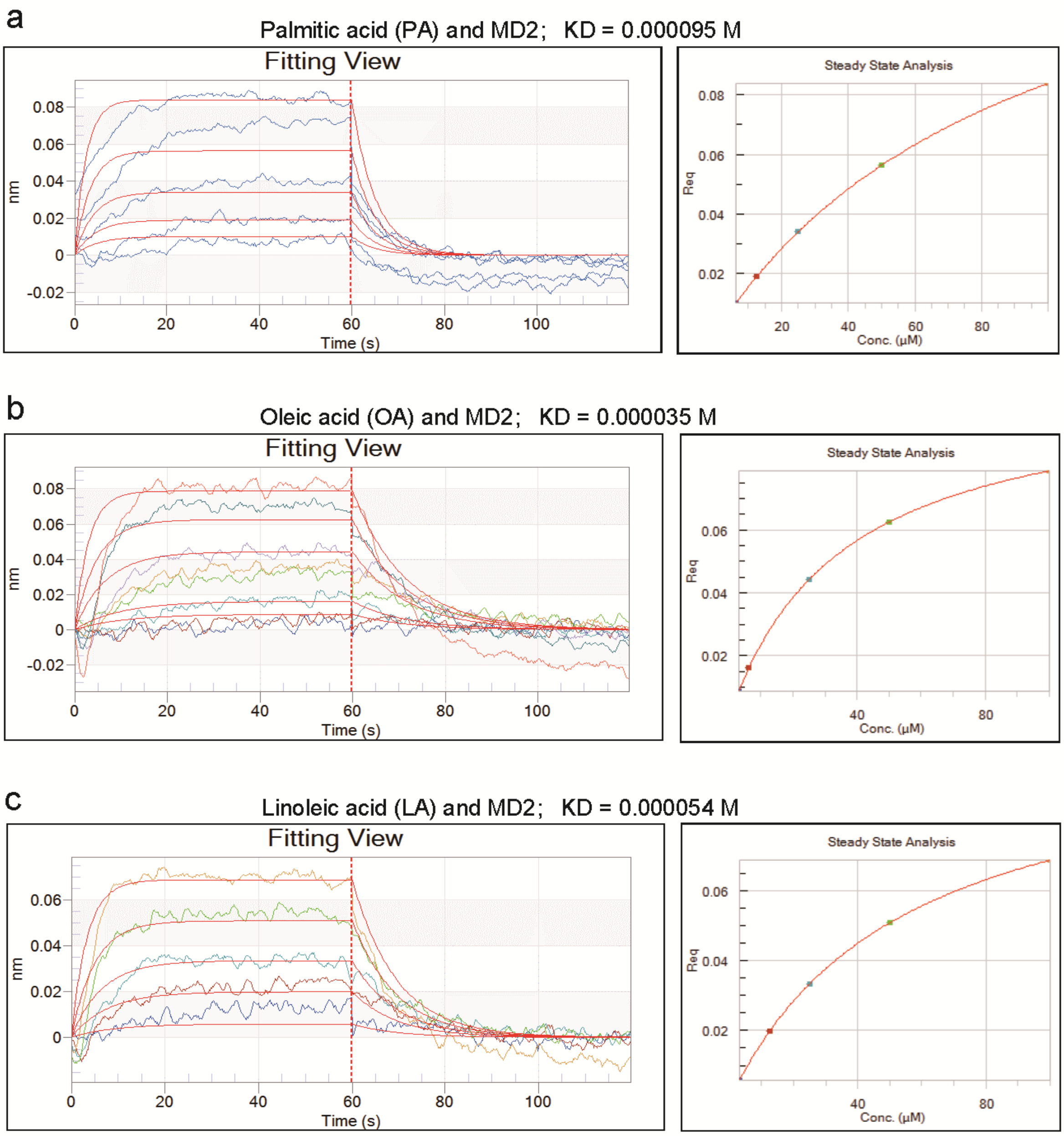

Supplementary Figure 17. MD2 binds unsaturated fatty acids.
Representative graphs from surface plasmon resonance spectroscopy analysis using ForteBio Octet HTX system (Pall Corporation, Menlo Park, CA) showing the binding of increasing concentrations of PA and unsaturated fatty acids (oleic and linoleic acids) with human recombinant MD2 (KD values are indicated on the upper of the panels).

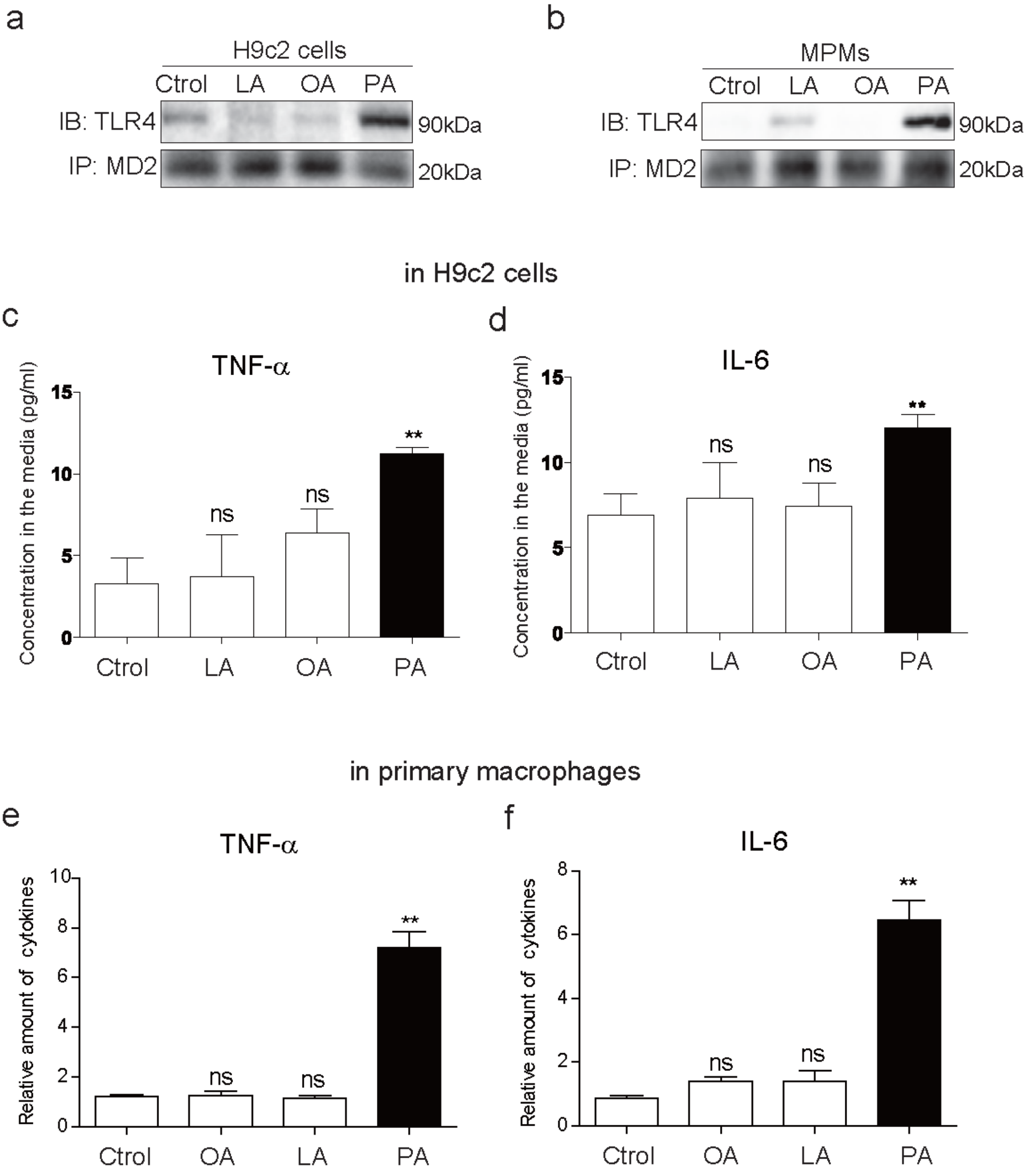

Supplementary Figure 18. Unsaturated fatty acids fail to induce MD2/TLR4 complex formation and induction of cytokines.

The ability of unsaturated fatty acids to induce MD2/TLR4 complex formation and pro-inflammatory cytokine production was assessed in H9C2 cells and primary macrophages. (**a** and **b**) Representative immunoprecipitation (IP) and Western blot (IB) analysis of MD2/TLR4 complex in H9C2 cells and macrophages (MPMs) treated with 100 µM PA, 100 µM linoleic acid (LNA), or 100 µM oleic acid (OA) for 15 min; n = 3. (**c**-**f**) Levels of TNF-α and IL-6 protein in culture media of H9C2 cells (b, c) and macrophages (d, e) following treatment with linoleic, oleic, and palmitic acids for 24 h; values reported as mean ± s.e.m. and analyzed by Student's *t*-test, $^{**}p<0.01$ compared to Ctrol; ns = not significant, n = 3.

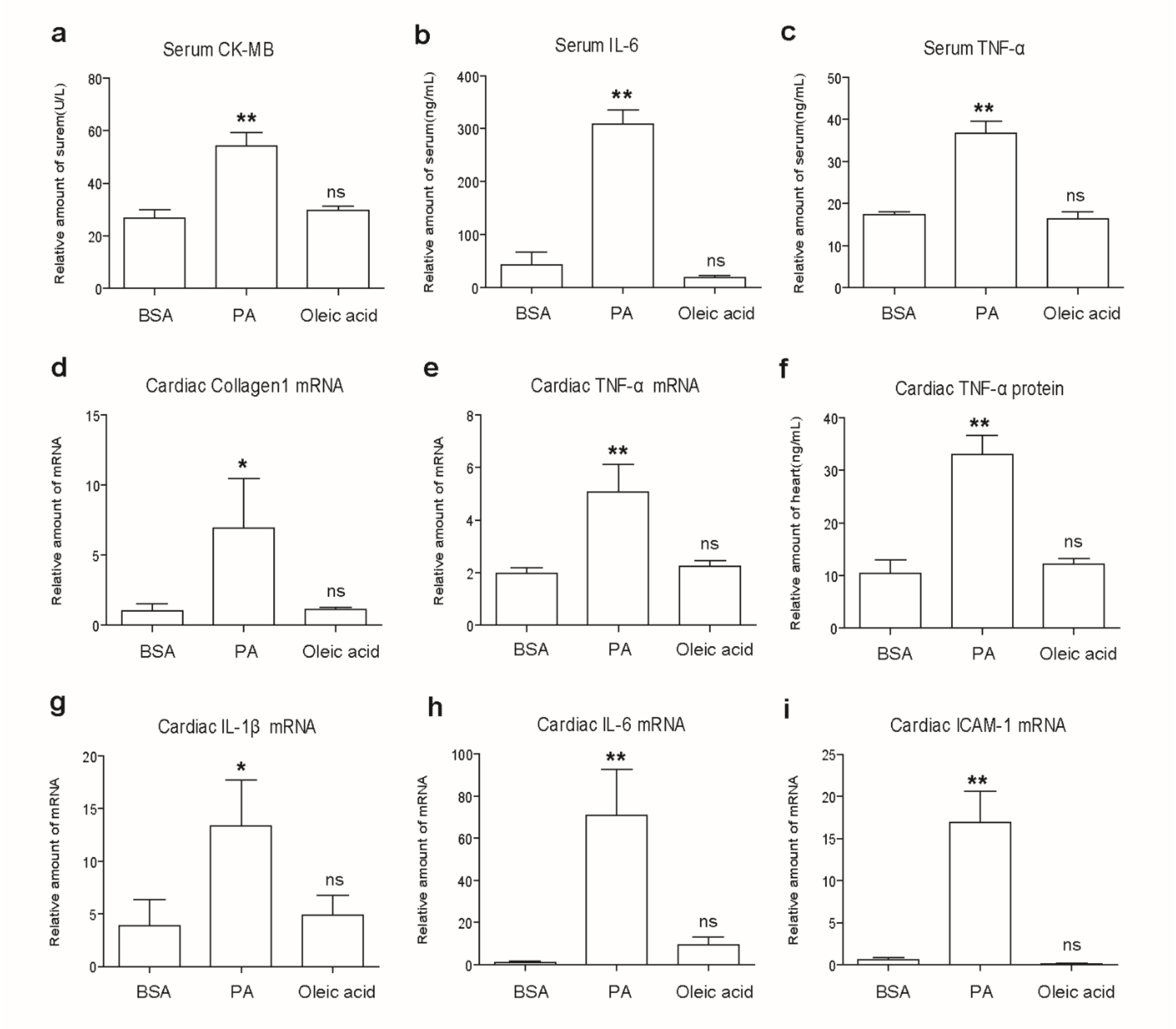

Supplementary Figure 19. Unsaturated fatty acid (oleic acid) does not induce myocardial injury in mice.

Wild-type (B6) mice were challenged with 5 mM PA, 5 mM oleic acid (OA) twice daily for 7 days by intravenous injection (n = 8). Control mice (Ctrl) received BSA vehicle control. Blood and heart tissues were collected for evaluation of inflammation and injury. (**a**-**c**) Serum levels of CK-MB (a, U L^{-1}), IL-6 (b, ng mL^{-1}), and TNF-α (c, ng mL^{-1}). (**d**-**h**) Cardiac tissue levels of collagen 1 mRNA (d), TNF-α mRNA (e), TNF-α protein (f). IL-1β mRNA (g), IL-6 mRNA (h), and ICAM-1 mRNA (i). Protein levels were measured by ELISA and mRNA levels by qPCR. Values reported as mean ± s.e.m. and analyzed by Student's *t*-test, *p<0.5, **p<0.01 compared to BSA, ns = not significant, n = 8.

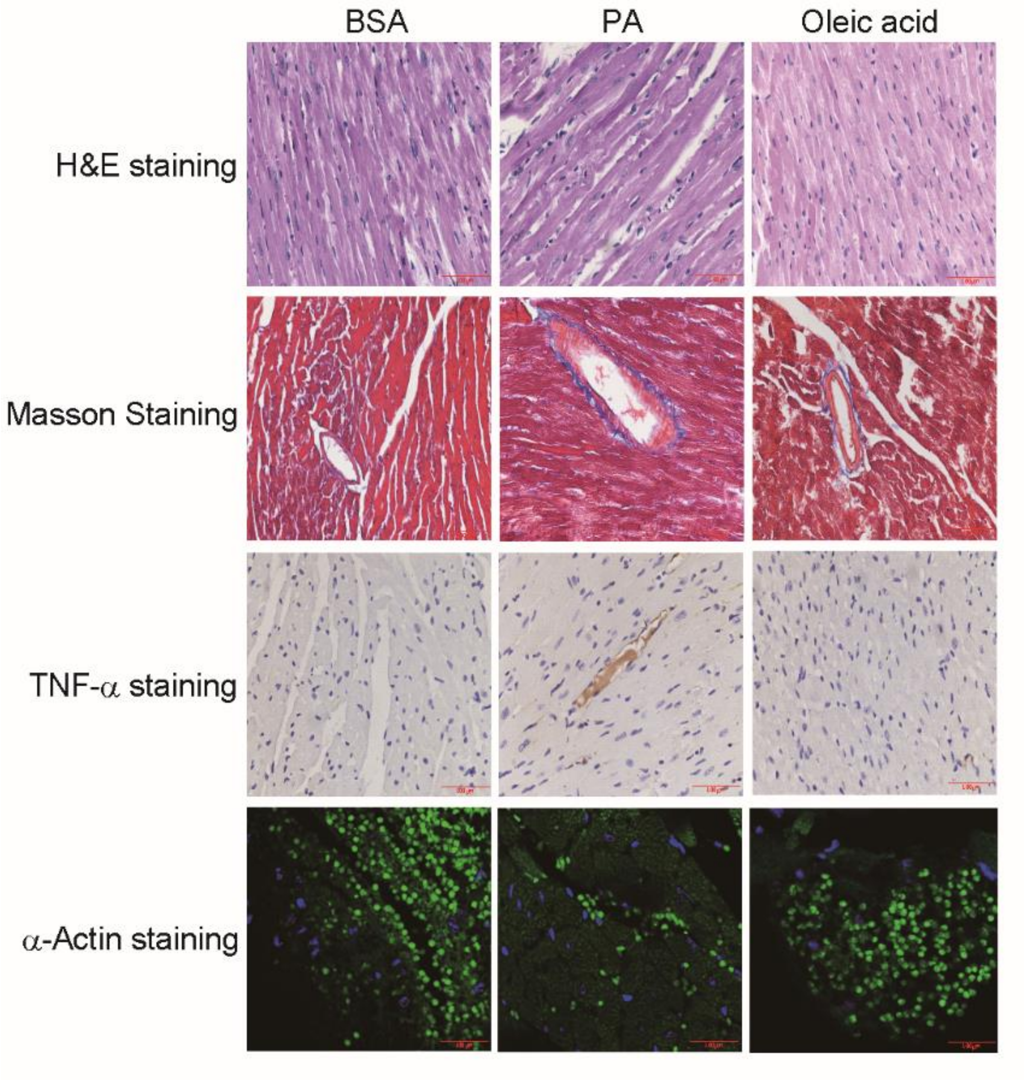

Supplementary Figure 20. Morphological analysis of cardiac tissues following oleic acid challenge.

Wild-type (B6) mice were challenged with 5 mM PA, 5 mM oleic acid (OA) twice daily for 7 days by intravenous injection (n = 8). Control mice (Ctrl) received BSA vehicle control. Representative images of heart tissues evaluated by H&E staining for structural changes, Masson's trichrome staining for fibrosis, TNF-α immunohistochemistry as index of inflammation, and α-actinin for cardiomyocytes, scale bars = 100 μm.

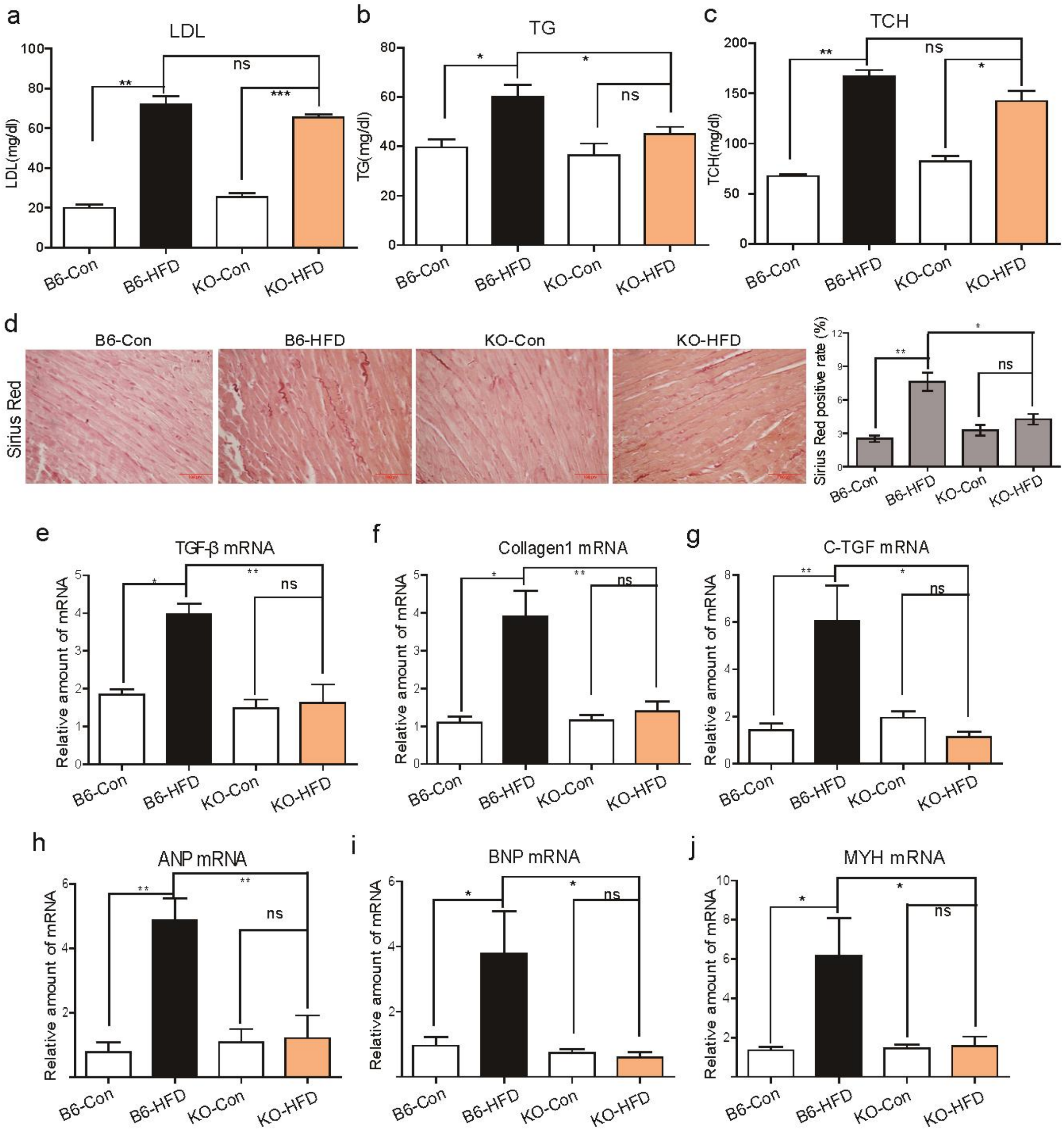

Supplementary Figure 21. ***Md2* knockout protects against inflammatory myocardial injuries in high-fat diet mouse model of obesity**

C57BL/6 (B6) or *Md2*$^{-/-}$ mice (KO) were fed a high fat diet (HFD) or normal control diet (Con) for 4 months. Blood and heart tissue were collected for evaluation. Shown are (**a**-**c**) circulating levels of low-density lipoproteins (LDL, a), triglycerides (TG, b), and total cholesterol (TCH, c). (**d**) Representative histochemical detection of fibrosis in heart tissue using Sirius Red staining (n = 8), with quantification of Sirius Red staining as index of fibrosis; scale bars = 100 μm. (**e**-**g**) Real-time qPCR analysis of TGF-β (e), Collagen 1 (f), and CTGF (g) mRNA in heart tissue. (**h**-**J**) mRNA analysis of hypertrophy markers in heart tissues showing levels of ANP (h), BNP (i), and MyHC (j). Data reported as mean ± s.e.m. analyzed by Student's *t*-test; * $p < 0.05$, ** $p < 0.01$, *** $p < 0.001$; compared with B6-HFD group; ns = not significant compared with KO-HFD group; n = 8.

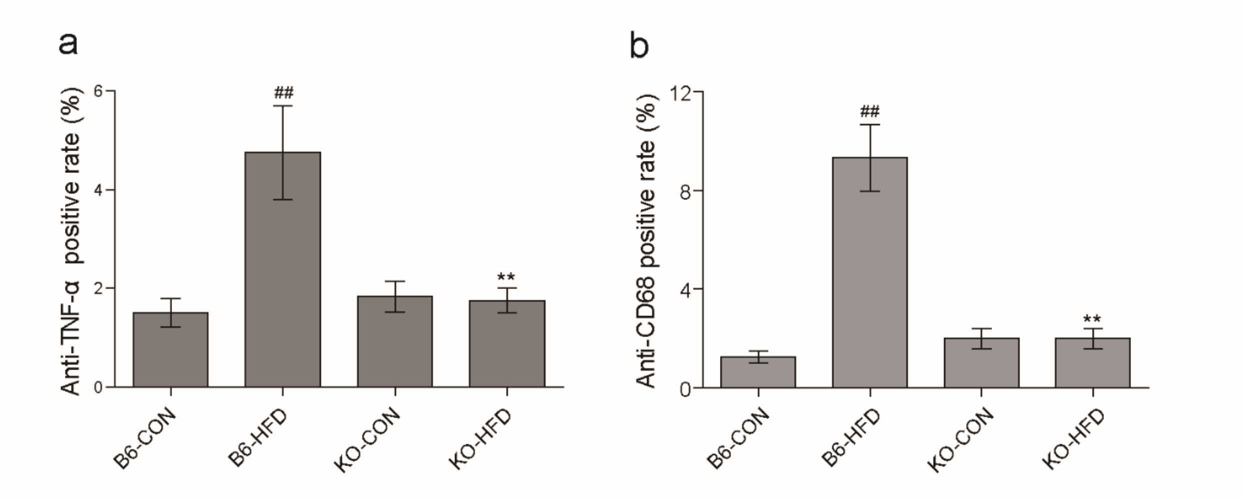

Supplementary Figure 22. Quantification of immunolocalization of TNF-α and CD68 presented in Figure 5k; values are reported as mean ± s.e.m. and analyzed by Student's *t*-test, ## $p < 0.01$ compared to B6-CON group; ** $p < 0.01$ compared to B6-HFD group; n = 8).

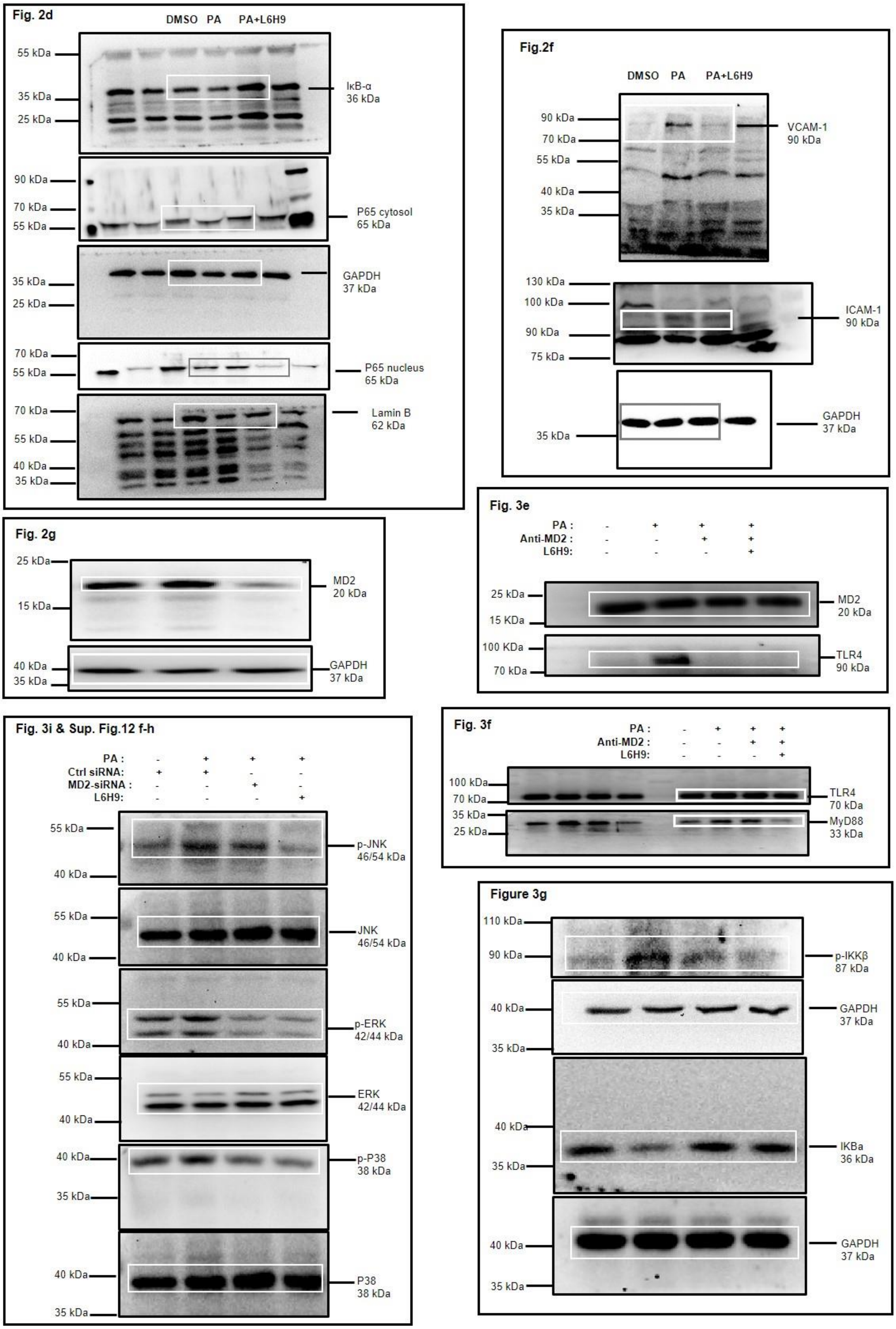

Supplementary Figure 23.

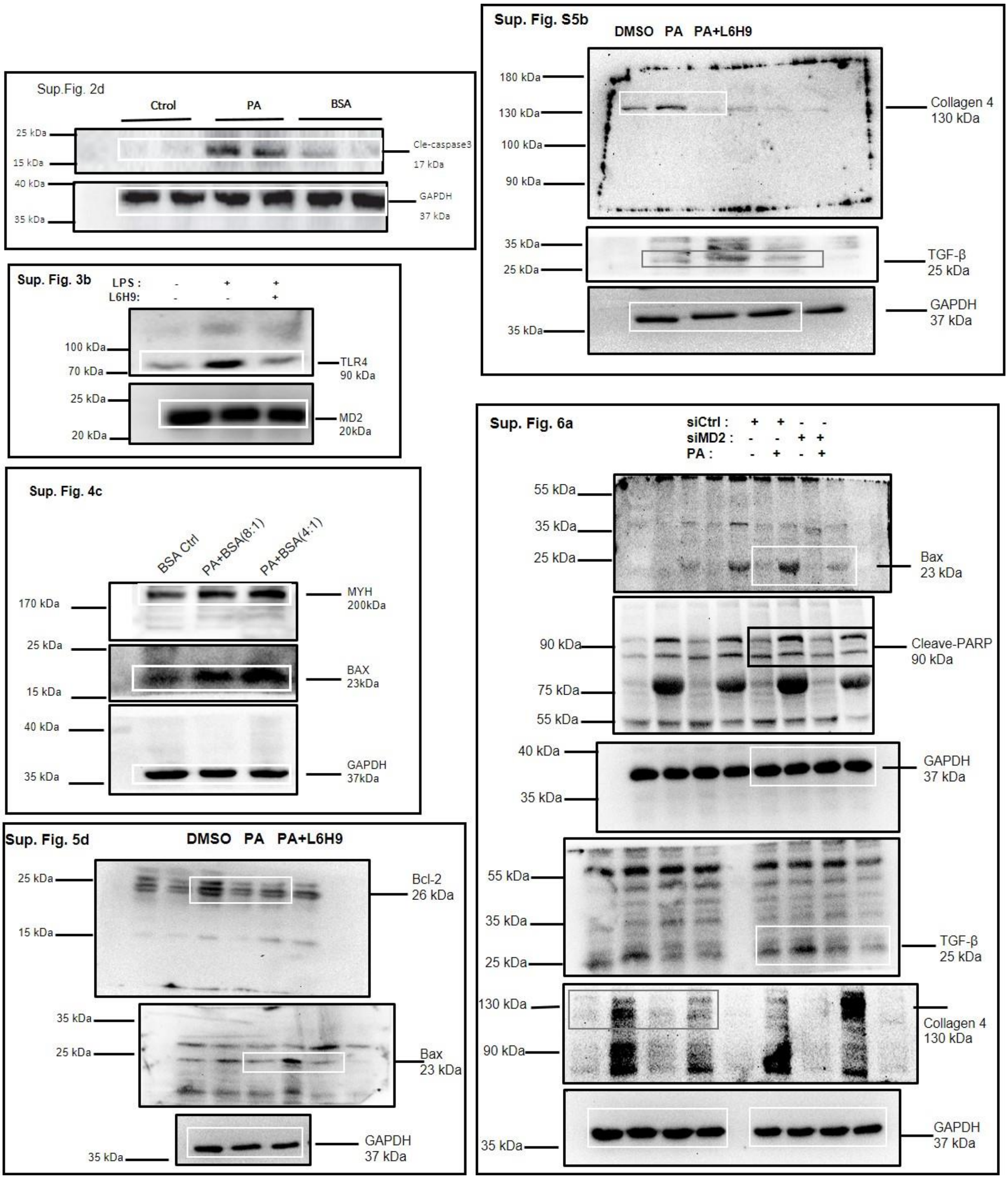

Supplementary Figure 23.

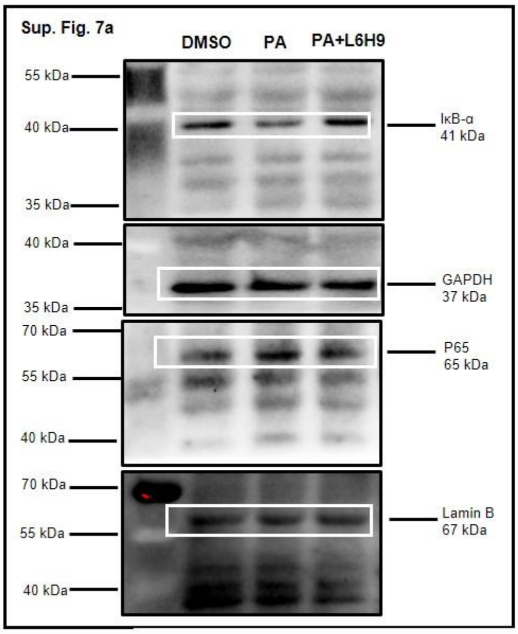

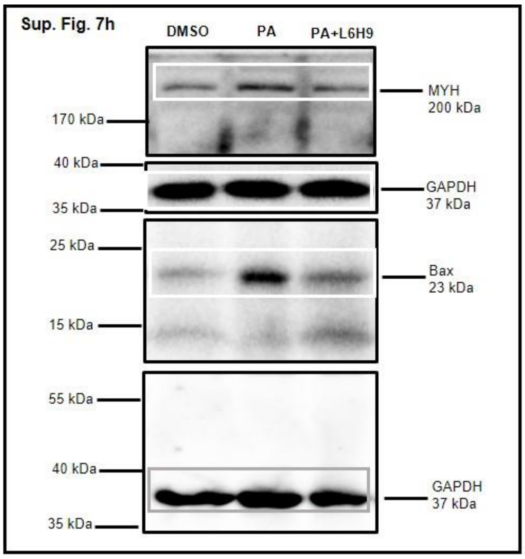

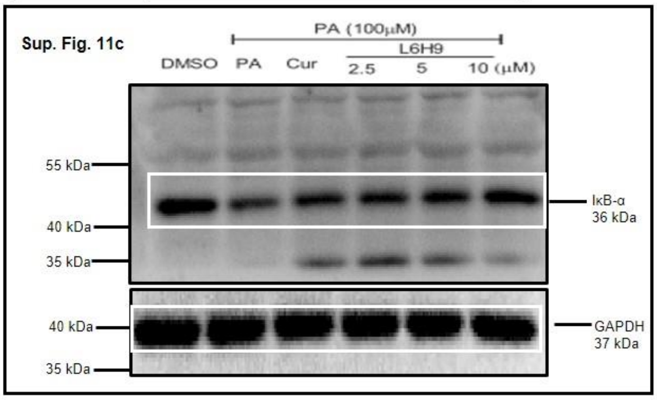

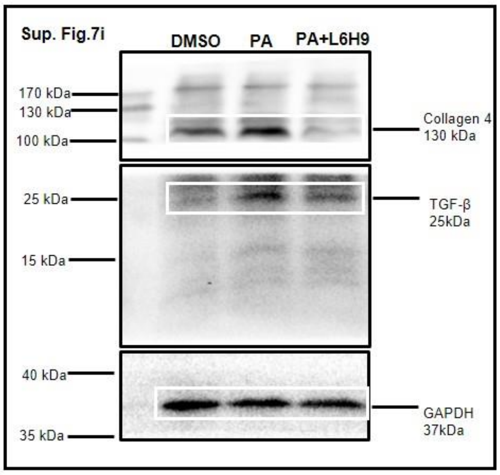

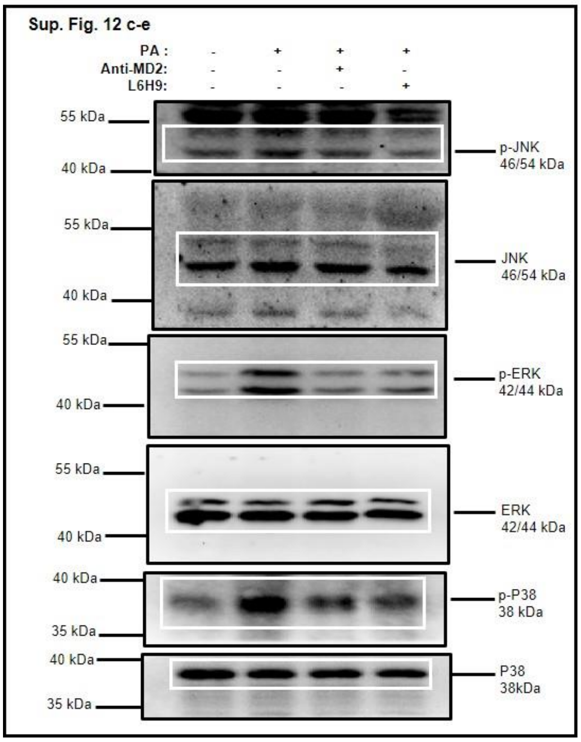

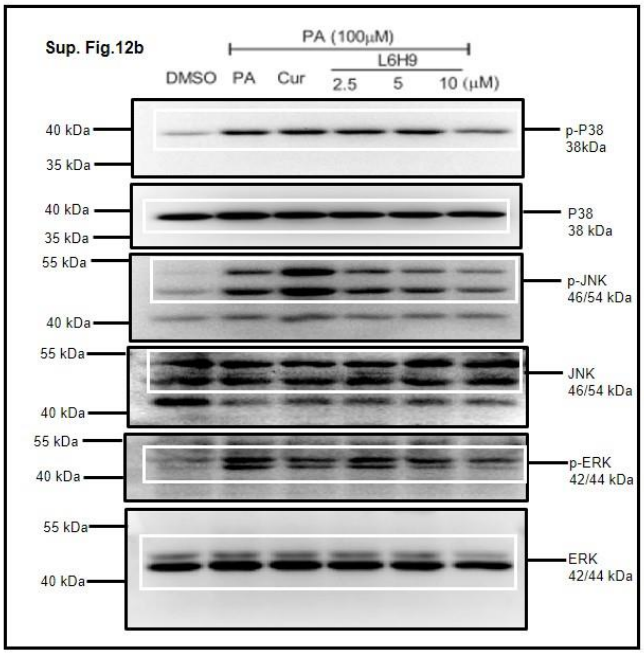

Supplementary Figure 23.

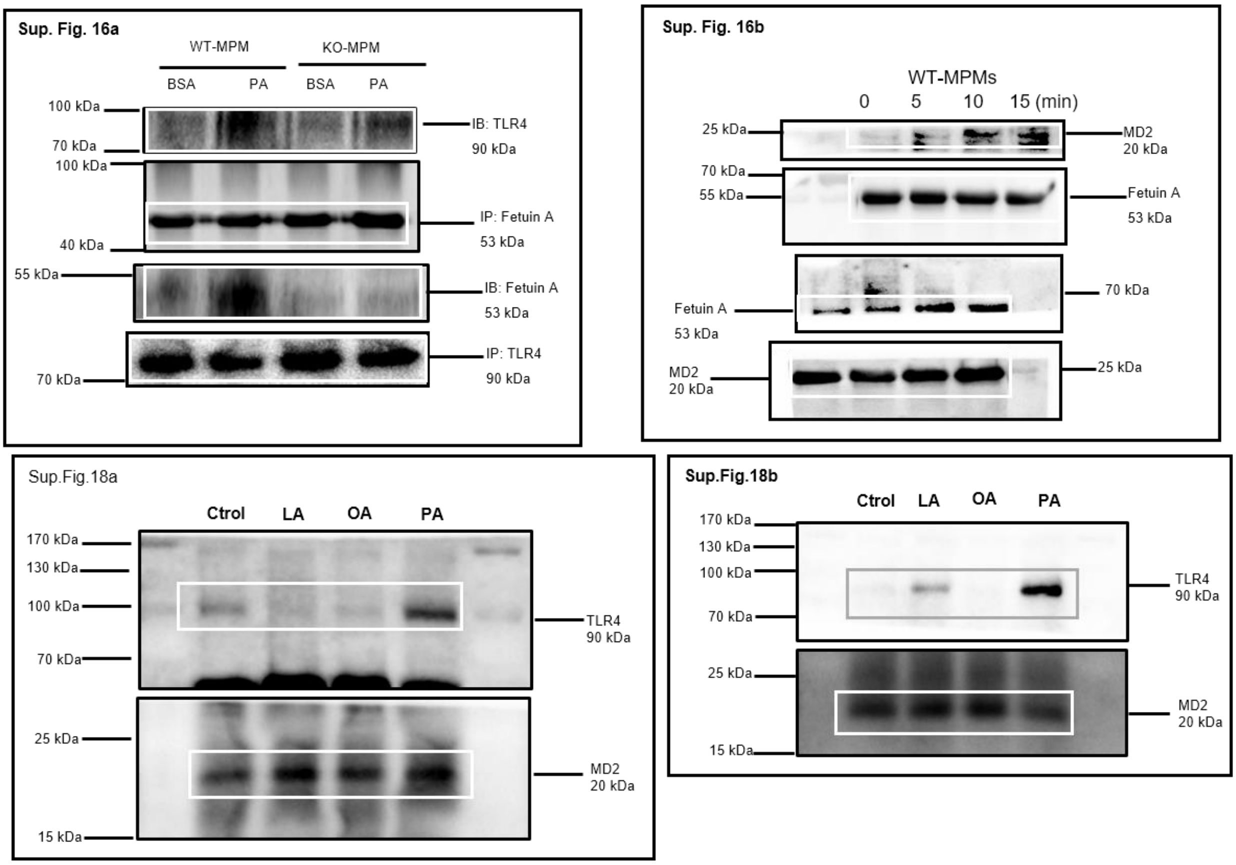

Supplementary Figure 23. Original un-cropped images of all the Western blots shown in the Figures and Supplementary Figures. The white or grey boxes indicate the area/bands selected for the figures. We used protein ladders (Thermo Scientific #26617) to identify the molecular weight of the protein of interest.
